# Exercise Programmes for People With Haemophilia: A Scoping Review

**DOI:** 10.1111/hae.70225

**Published:** 2026-02-20

**Authors:** Maísa Veríssimo, Tatiana Kuhn, Janaina Ricciardi, Carolina Kosour, Mônica Veríssimo, Flávia Maia, Olga Ribeiro, Renata C. Gasparino

**Affiliations:** ^1^ Graduate Programme in Nursing University of Campinas (UNICAMP) Campinas São Paulo Brazil; ^2^ Fundação Centro Médico de Campinas Campinas São Paulo Brazil; ^3^ Haemophilia Treatment Centre (HTC) “Cláudio Luiz Pizzigatti Corrêa” University of Campinas (UNICAMP) São Paulo Brazil; ^4^ Federal University of Alfenas (UNIFAL‐MG) Alfenas Minas Gerais Brazil; ^5^ Centro Infantil de Investigações Hematológicas Dr. Domingos A. Boldrini Campinas São Paulo Brazil; ^6^ JBI Universidade Estadual de Campinas Evidence Based Healthcare Research Centre School of Nursing University of Campinas (UNICAMP) Campinas São Paulo Brazil; ^7^ RISE‐Health Nursing School University of Porto Porto Portugal; ^8^ School of Nursing University of Campinas (UNICAMP) Campinas São Paulo Brazil

**Keywords:** exercise, exercise therapy, haemophilia, physical activity, scoping review

## Abstract

**Background:**

Advances in haemophilia treatment have enabled safe exercise practice as recommended by disease management guidelines, yet there is no gold‐standard protocol for optimal dose. Emerging therapies could influence exercise recommendations, highlighting the need for evidence‐based guidelines tailored to people with haemophilia to ensure better safety and health outcomes.

**Aims:**

To provide an updated comprehensive mapping of the literature, exploring the modalities, frequency, duration and intensity of exercise programmes for PwH.

**Methods:**

A scoping review was conducted following JBI methodology and PRISMA‐ScR guidelines (PubMed, BVS, Scopus, Web of Science, EMBASE, Cochrane, PEDro, SPORTDiscus and grey literature). Inclusion criteria were defined using ‘PCC’ (population, concept and context), and the research question was ‘What modalities, duration, frequency and intensity are being utilised in exercise programmes for PwH in any context?’

**Results:**

Out of 5.579 references, 36 studies were included, with 15 reported in a single source. Exercise frequency was mainly set at 3 days/week in primary studies, with a median of 2–4 days/week reported in systematic reviews. Training intensity was mainly defined by repetition maximum or maximum heart rate. Interventions generally lasted 6 weeks, with a median duration of 4–30 weeks in systematic reviews. Strengthening, flexibility, and aerobic exercise were the most common modalities, whereas endurance and proprioceptive training were rarely employed.

**Conclusions:**

Exercise dosages in this review align with current evidence for people with haemophilia, but individualised prescriptions remain critical to optimising health outcomes. Future research should prioritise standardised, evidence‐based protocols.

## 1 Introduction

Historically, people with haemophilia (PwH) were advised against physical activity (PA) due to concerns about bleeding risks [1]. However, the advent of clotting factor replacement therapy and the shift towards prophylactic treatment regimens, consisting of regular administration of therapeutic products to maintain haemostasis and prevent acute haemorrhagic episodes, have changed this paradigm. This allowed PwH to safely engage in regular PA, an essential component of managing their condition, as recommended by recent guidelines [2, 3].

Although PA and exercise are often used interchangeably, they are not synonymous. PA is defined as any movement produced by muscle contraction that results in energy expenditure, whereas exercise is a subset of PA that consists of planned, systematic, and repetitive movement to improve or maintain one or more aspects of physical fitness [4].

Exercise as a treatment for PwH has shown numerous benefits and is considered safe, although there is no recommended gold‐standard protocol [5]. When correctly prescribed and managed, it is highly beneficial in maintaining musculoskeletal health, reducing pain perception and bleeding frequency, as well as complications associated with hemarthrosis, including chronic arthropathy and functional impairment [2, 6].

Despite the clear benefits of exercise, there is no universally accepted protocol for its prescription in PwH. An emblematic systematic review of the safety and efficacy of exercise in haemophilia was published by Cochrane in 2016 [5]. Subsequent reviews published since 2020 have focused either on assessing the safety and effectiveness of specific exercise modalities [7, 8] or their impact on a single health‐related outcome [9], rather than providing a panorama of exercise modalities available for this population.

The treatment landscape for haemophilia has evolved significantly in recent years, with several advances that could influence how we approach exercise prescription in PwH. One important development is the rise of non‐factor replacement therapies, such as *emicizumab* for haemophilia A and gene therapy trials for both haemophilia A and B [3]. These innovative treatments have the potential to reduce joint bleeding rates and, consequently, the onset of joint deterioration and degenerative disease, which could impact exercise recommendations by potentially allowing for more intense or frequent PA.

This study aims to provide an updated comprehensive mapping of the literature, with a scoping review as a potential approach to address the nature and diversity of the knowledge available, exploring the modalities, frequency, duration and intensity of exercise programmes for PwH. By providing a detailed overview of current practices, this review will offer valuable insights for clinicians and researchers while identifying areas where standardised protocols may be developed.

## 2 Methods

This review was conducted following the JBI methodology [10] for scoping reviews, and the Preferred Reporting Items for Systematic Reviews and Meta‐Analyses extension for Scoping Reviews (PRISMA‐ScR) [11]. The protocol has been registered in OSF and can be accessed at https://osf.io/6ujfc [12].

### 2.1 Inclusion Criteria

The inclusion criteria were defined using the mnemonic ‘PCC’ (population, concept and context):

Population: People of any age with mild, moderate or severe type A or B haemophilia, regardless of their treatment regimen (except gene therapy). Haemophilia A and B are X‐linked bleeding disorders caused by a deficiency of clotting factor VIII or factor IX, respectively, characterised by the level of factor activity as severe (<0.01 IU/mL), moderate (0.01–0.05 IU/mL) or mild (0.05–0.40 IU/mL), which typically correlates with the severity of bleeding symptoms [3].

Concept: Supervised or unsupervised exercise programmes for prevention or rehabilitation purposes. The exercise modalities included were selected based on the MeSH glossary definitions for the descriptors ‘Exercise’, ‘Exercise Therapy’ and ‘Exercise Movement Techniques’ and respective sub‐descriptors, including Aerobic Exercise, Aquatic Exercise/Hydrotherapy, Balance Training, Breathing Exercises, Circuit‐Based Exercise, Continuous Passive Movement (CPM), Dance Therapy, Endurance Training, Exergaming, Gymnastics, High‐Intensity Interval Training, Muscle Stretching Exercises, Plyometric Exercise, Proprioceptive Exercise, Resistance Training, Running, Swimming, Tai Ji, Walking and Yoga [13]. Sources were excluded if the interventions were exercise tests, sports, did not report exercise dosage, or lacked pre‐ or post‐intervention outcomes. Sources reporting exercise programmes paired with other physiotherapy interventions were excluded if it was not possible to extract the exercise parameters individually. As the aim of this review was map these parameters, studies that did not include a group receiving solely exercise were not eligible.

Context: Interventions delivered in any settings, including primary, secondary or tertiary healthcare settings, home‐based or community‐based.

### 2.2 Types of Sources

To comprehensively map exercise interventions for PwH, published and unpublished sources in any language were included, with no restrictions on study design or publication date (up to April 5th, 2024). Grey literature was also included to capture relevant data that may not have been available in peer‐reviewed publications, providing a broader view of interventions.

### 2.3 Search Strategy

A three‐stage search strategy was employed. First, an initial search of the MEDLINE (PubMed) and Scopus databases was conducted to identify available publications. The titles, abstracts, and index terms found were used to develop the full search strategy (see Appendix S1), which was adapted and applied to: MEDLINE (PubMed and PubMed Central), BIREME (BVS), Scopus, Web of Science, EMBASE (EBSCOhost), Cochrane, PEDro and SPORTDiscus (EBSCOhost). Grey literature was searched using Google Scholar, the Brazilian Digital Library of Theses and Dissertations (BDTD), the ProQuest Dissertations & Theses Database, and the websites of haemophilia organisations [14, 15, 16, 17, 18]. The reference lists of publications included in this review were also screened for additional sources.

### 2.4 Study Selection

All identified sources were exported to Rayyan, and duplicates were removed. Three reviewers (M.P.A.V., T.N.K. and R.C.G.) conducted a pilot test screening of a random sample of 10 sources. After pilot‐testing, two independent reviewers (M.P.A.V. and T.N.K.) screened the titles and abstracts. Publications were excluded if the full text could not be retrieved or if they could not be translated using free tools, such as DeepL and Google Translate. To ensure the accuracy of the translations, more than one tool was used, and the translated versions were compared to one another. Where possible, the translated versions were checked by reviewers fluent in the source languages. The relevant publications were then retrieved in full and independently assessed by the same reviewers (M.P.A.V. and T.N.K.). Conflicts were identified by the management software and disagreements in the screening process were initially resolved through discussion between the independent reviewers (M.P.A.V. and T.N.K.). Where necessary, a third reviewer (R.C.G.) was involved to render a final decision, maintaining the impartiality of the process.

### 2.5 Data Extraction and Synthesis

A data extraction tool developed for this review, based on JBI recommendations, was used. The extraction was performed by M.P.A.V. and reviewed by T.N.K., with adjustments made to the tool as necessary throughout this phase. In accordance with the JBI framework, critical appraisal of the methodological quality was not conducted [10, 19]. The data are presented in Tables 1, 2, 3, providing an overview of the included studies, participants’ characteristics and exercise parameters. The results of the interventions, including statistical significance presented in the respective papers, are summarised in Appendix S3.

**TABLE 1:** Overview of included studies (*n* = 36).

Author (year)	Country	Design	Journal	Title	Context
Pelletier et al. (1987) [28]	United States	Case report	Physical therapy	Isometric exercise for an individual with hemophilic arthropathy	Clinical
Tiktinsky et al. (2002) [20]	Israel	Prospective and retrospective pilot	Haemophilia	The effect of resistance training on the frequency of bleeding in haemophilia patients: A pilot study	Clinical
Gomis et al. (2009) [48]	Spain	Systematic review	Haemophilia	Exercise and sport in the treatment of haemophilic patients: A systematic review	Clinical and non‐clinical
Vallejo et al. (2010) [30]	Spain	Single‐arm prospective	Haemophilia	Influence of aquatic training on the motor performance of patients with haemophilic arthropathy	Clinical
Mulvany et al. (2010) [25]	United States	Single arm pre‐test‐post‐test	Physical therapy	Effects of a 6‐week, individualized, supervised exercise program for people with bleeding disorders and hemophilic arthritis	Clinical
Querol et al. (2011) [50]	Spain	Systematic review	Apunts Medicina de l'Esport	Hemofilia: Ejercicio y deporte	Clinical and non‐clinical
Souza et al. (2012) [49]	Brazil	Systematic review	International Journal of Sports Medicine	Haemophilia and exercise	Clinical
Kargarfard et al. (2013) [23]	Iran	Non‐RCT	International Journal of Preventive Medicine	The effect of aquatic exercise therapy on muscle strength and joint's range of motion in hemophilia patients	Clinical
Eid et al. (2014) [22]	Egypt	RCT	The Egyptian Journal of Medical Human Genetics	Effect of resistance and aerobic exercises on bone mineral density, muscle strength and functional ability in children with hemophilia	Clinical
Parhampour et al. (2014) [26]	Iran	RCT	Clinical Rehabilitation	Effects of short‐term resistance training and pulsed electromagnetic fields on bone metabolism and joint function in severe haemophilia A patients with osteoporosis: A randomized controlled trial	Clinical
Al‐Sharif et al. (2014) [21]	Saudi Arabia	RCT	African Health Sciences	Impact of mild versus moderate intensity aerobic walking exercise training on markers of bone metabolism and hand grip strength in moderate hemophilic a patients	Clinical
Mohamed & Sherief (2015) [24]	Egypt	RCT	The Egyptian Journal of Medical Human Genetics	Bicycle ergometer versus treadmill on balance and gait parameters in children with hemophilia	Clinical
Cruz et al. (2015) [32]	Brazil	Case report	Acta Fisiátrica	Resultados de um programa de condicionamento físico em um paciente com hemofilia A grave	Clinical
Blum (2015) [47]	Australia	Systematic review	Aqualines	The effectiveness of aquatic physiotherapy in patients with haemophilia: A review of the literature	Clinical
Runkel et al. (2016) [29]	Germany	RCT	Haemophilia	RCT of a 6‐month programmed sports therapy (PST) in patients with haemophilia—Improvement of physical fitness	Non‐clinical (qualified fitness studios/corresponding local institution)
Strike et al. (2016) [5]	Canada	Systematic review	Cochrane Database of Systematic Reviews	Exercise for haemophilia	Clinical
Schäfer et al. (2016) [51]	Brazil	Systematic review	Haemophilia	Physical exercise, pain and musculoskeletal function in patients with haemophilia: A systematic review	Clinical and non‐clinical
Runkel et al. (2017) [43]	Germany	RCT	Haemophilia	RCT—subjective physical performance and quality of life after a 6‐month programmed sports therapy (PST) in patients with haemophilia	Non‐clinical (qualified fitness studios/corresponding local institution)
Parhampour et al. (2019) [27]	Iran	RCT	Haemophilia	The effects of six‐week resistance, aerobic and combined exercises on the pro‐inflammatory and anti‐inflammatory markers in overweight patients with moderate haemophilia A: A randomized controlled trial	Clinical
Neelapala et al. (2019) [46]	India	Systematic review	Complementary Therapies in Clinical Practice	Aquatic exercise for persons with haemophilia: A review of literature	Clinical
Deniz and Güzel (2020) [34]	Turkey	RCT	International Journal of Disabilities Sports and Health Sciences	Do therapeutic exercises improve kinesophobia and health‐related quality of life in adult hemophilia patients? A randomized controlled trial	Non‐clinical (gymnasium)
Calatayud et al. (2020) [31]	Spain	RCT	Physical Therapy	Safety and effectiveness of progressive moderate‐to‐vigorous intensity elastic resistance training on physical function and pain in people with hemophilia	Clinical
Wagner et al. (2020) [7]	Austria	Systematic review	Haemophilia	The effect of resistance exercise on strength and safety outcome for people with haemophilia: A systematic review	Clinical and non‐clinical
García‐Dasí et al. (2021) [37]	Spain	Two‐arm prospective	Haemophilia	Effects of a non‐pharmacological approach for chronic pain management in patients with haemophilia: Efficacy of cognitive‐behavioural therapy associated with physiotherapy	Non‐clinical (home‐based)
Parhampour et al. (2021) [40]	Iran	RCT	Medical Journal of the Islamic Republic of Iran	Effects of short‐term aerobic, resistance and combined exercises on the lipid profiles and quality of life in overweight individuals with moderate hemophilia A: A randomized controlled trial	Non‐clinical (sports facility)
Pillard et al. (2021) [42]	France	Exploratory	Science and Sports	Non‐clinical and personalized endurance training program for patients with mild to moderate hemophilia A: What can be expected?	Non‐clinical (home‐based)
Parhampour et al. (2022) [41]	Iran	RCT	Haemophilia	Muscle thickness and pennation angle in overweight persons with moderate haemophilia A after resistance and combined training: A randomized controlled trial	Clinical
Gönen et al. (2022) [38]	Turkey	RCT	Haemophilia	The effects of close kinetic chain exercises on proprioception and physical activity level in pediatric patients with hemophilia	Clinical
Fares et al. (2022) [36]	Saudi Arabia	RCT	European Review for Medical and Pharmacological Sciences	The efficacy of aerobic training on the pulmonary functions of hemophilic A patients: A randomized controlled trial	Clinical
Tomschi et al. (2022) [8]	Germany	Systematic review	Haemophilia	Aerobic exercise in patients with haemophilia: A systematic review on safety, feasibility and health effects	Clinical
Chimeno‐Hernández et al. (2022) [9]	Spain	Systematic review	Haemophilia	Effectiveness of physical exercise on postural balance in patients with haemophilia: A systematic review	Clinical and non‐clinical
Cruz‐Montecinos et al. (2023) [52]	Spain	RCT	European Journal of Haematology	Effectiveness of progressive moderate‐vigorous intensity elastic resistance training on quality of life and perceived functional abilities in people with hemophilia: Secondary analysis of a randomized controlled trial	Clinical
Moreno‐Segura et al. (2023) [39]	Spain	RCT	Haemophilia	Effectiveness of therapeutic exercise and cognitive‐behavioural therapy combined protocol on functionality, pain and joint health in people with haemophilia: Secondary analysis of a controlled trial	Non‐clinical (home‐based)
Wilczyński et al. (2023) [45]	Poland	Case report	Physiotherapy Theory and Practice	Strength training program for an athlete with hemophilia A and an inhibitor while taking a new prophylactic drug treatment: A case report	Clinical
Elnaggar et al. (2024) [35]	Saudi Arabia	RCT	Physical and Occupational Therapy in Pediatrics	Effects of plyometric‐based hydro‐kinesiotherapy on pain, muscle strength, postural stability, and functional performance in children with hemophilic knee arthropathy: A randomized trial	Clinical
Srichumpuang et al. (2024) [44]	Thailand	Non‐RCT	Orphanet Journal of Rare Diseases	Moderate‐ to vigorous‐intensity physical activities for hemophilia A patients during low‐dose pharmacokinetic‐guided extended half‐life factor VIII prophylaxis	Clinical and non‐clinical

Abbreviation: RTC, randomized clinical trial.

**TABLE 2:** Overview of participants’ characteristics (*n* = 36).

Author (year) Design	Sample	Age	Haemophilia severity	Factor replacement therapy
Pelletier et al. (1987) [28] Case report	1	12	Severe	Factor VIII replacement during intervention
Tiktinsky et al. (2002) [20] Prospective and retrospective pilot	2	P1: 26 P2: 37	Severe (2)	On demand (2)
Gomis et al. (2009) [48] Systematic review	N/A	N/A	Severe (7) N/A (22)	Prophylaxis (6) On demand (3) N/A (13)
Vallejo et al. (2010) [30] Single‐arm prospective	13^*^ ^*^9 have completed the intervention	32.27 ± 1.5	Moderate (1) Severe (12)	Prophylaxis (4) On demand (9)
Mulvany et al. (2010) [25] Single arm pre‐test‐post‐test	33^*^, ^**^ ^*^20 have completed the intervention ^**^3 with Von Willebrand's disease	7–57	Mild (1) Moderate (3) Severe (26)	Participants with severe haemophilia received a prophylactic dose of factor before exercise (26)
Querol et al. (2011) [50] Systematic review	N/A	N/A	Severe (7) N/A (11)	N/A
Souza et al. (2012) [49] Systematic review	152	N/A	Severe (3) Mild/Moderate (1) Moderate/Severe (1) Mild/Moderate/Severe (3)	N/A
Kargarfard et al. (2013) [23] Non‐RCT	20 IG: 10 CG: 10	IG: 22.9 ± 7.6 CG: 18.1 ± 6.26	Moderate (20)	N/A
Eid et al. (2014) [22] RCT	30 IG: 15 CG: 15	IG: 12 ± 1.36 CG: 12.13 ± 1.35	Moderate (30)	Recombinant factor VIII replacement (30)
Parhampour et al. (2014) [26] RCT	48 RT: 13^*^ RTPEMF: 12^*^ PEMF: 11 CG: 12 ^*^10 have completed the intervention	20–35	Severe (48)	Prophylaxis (48)
Al‐Sharif et al. (2014) [21] RCT	50 A: 25 B: 25	A: 38.14 ± 8.32 B: 37.97 ± 9.15	Moderate (50)	On demand (50)
Mohamed and Sherief (2015) [24] RCT	30 A: 15 B: 15	A: 12.4 ± 1.37 B: 12.8 ± 1.39	Mild to Moderate (30)	On demand (30)
Cruz et al. (2015) [32] Case report	1	31	Severe	Prophylaxis
Blum (2015) [47] Systematic review	91	N/A	N/A	N/A
Runkel et al. (2016) [29] RCT	64 IG: 32^*^ CG: 32^**^ ^*^24 included in data analysis ^**^28 included in data analysis	IG: 41.9 ± 10.6 CG: 40.3 ± 8.8	Moderate (5) Severe (59)	Prophylaxis (IG: 92%; CG: 86%) On demand (IG: 0; CG: 7%) N/A (IG: 8%; CG: 7%)
Strike et al. (2016) [5] Systematic review	233	8–49	Moderate (4) Severe (1) Mild/Severe (1) Mild/Moderate/Severe (2)	Factor infusion pre‐intervention (2)
Schäfer et al. (2016) [51] Systematic review	294	25–64	Moderate (1) Severe (2) N/A (6)	Prophylaxis (9)
Runkel et al. (2017) [43] RCT	64 IG: 32^*^ CG: 32^**^ ^*^24 included in data analysis ^**^28 included in data analysis	IG: 41.9 ± 10.6 CG: 40.3 ± 8.8	Moderate (IG: 3; CG: 2) Severe (IG: 21; CG: 26)	N/A
Parhampour et al. (2019) [27] RCT	48 RT: 12 AT: 12 CT: 12 CG: 12	RT: 46.42 ± 4.68 AT: 45.83 ± 5.37 CT: 45.5 ± 6.58 CG: 45.17 ± 4.74	Moderate (48)	Prophylaxis (48)
Neelapala et al. (2019) [46] Systematic review	79	N/A	Moderate (1)	N/A
Deniz and Güzel (2020) [34] RCT	24 IG: 12 CG: 12	IG: 26.3 ± 6.6 CG: 25.5 ± 8.8	Moderate (IG: 4; CG: 3) Severe (IG: 8; CG: 9)	N/A
Calatayud et al. (2020) [31] RCT	20 IG: 10 CG: 10	IG: 36.3 ± 10.5 CG: 39.1 ± 8.4	Mild (IG: 1; CG: 1) Moderate (IG: 0; CG: 1) Severe (IG: 9; CG: 8)	Prophylaxis (IG: 9; CG: 8) On demand (IG: 1; CG: 2)
Wagner et al. (2020) [7] Systematic review	461^*^ ^*^including people with haemophilia and other coagulopathies	7–66	Moderate (3) Severe (2) Mild/Severe (1) Moderate/Severe (5) Mild/Moderate/Severe (3)	Prophylaxis (3) On demand (2) Prophylaxis and on demand (2) Prophylaxis or on demand (4) N/A (3)
García‐Dasí et al. (2021) [37] Two‐arm prospective	19 IG: 10 CG: 9	IG: 45 ± 8.56 CG: 37.89 ± 13.31	Severe (19)	Prophylaxis (19)
Parhampour et al. (2021) [40] RCT	60 RT: 15 AT: 15 CT: 15 CG: 15	RT: 46.87 ± 4.27 AT: 46.2 ± 5.59 CT: 46 ± 6.07 CG: 46.07 ± 4.68	Moderate (60)	Prophylaxis (60)
Pillard et al. (2021) [42] Exploratory	17	35 ± 7.6	Mild (13) Moderate (4)	N/A
Parhampour et al. (2022) [41] RCT	42 RT: 14 CT: 14 CG: 14	RT: 46.79 ± 4.42 CT: 46 ± 6.3 CG: 45.71 ± 4.64	Moderate (42)	Prophylaxis (42)
Gönen et al. (2022) [38] RCT	24 IG: 8^*^ CTG: 8^*^ CG: 8^*^ ^*^7 included in data analysis	IG: 11.43 ± 1.81 CTG: 13 ± 3.06 CG: 11.14 ± 2.41	Moderate (6) Severe (15)	Prophylaxis (24)
Fares et al. (2022) [36] RCT	40 IG: 20 CG: 20	IG: 28.3 ± 3.11 CG: 27.8 ± 4.29	Mild (IG: 14; CG: 12) Moderate (IG: 6; CG: 8)	Prophylaxis (40)
Tomschi et al. (2022) [8] Systematic review	193 IG: 91 CG: 102	10–83	Moderate (6) Severe (15)	Prophylaxis (86) On demand (73)
Chimeno‐Hernández et al. (2022) [9] Systematic review	304	9–59	Mild (0.66%) Moderate (22.7%) Severe (48.35%) Mild/Moderate (9.87%) Moderate/Severe (18.72%)	Prophylaxis (204) On demand (64) N/A (36)
Cruz‐Montecinos et al. (2023) [52] RCT	20 IG: 10 CG: 10	IG: 36.3 ± 10.5 CG: 39.1 ± 8.4	Mild (IG: 1; CG: 1) Moderate (IG: 0; CG: 1) Severe (IG: 9; CG: 8)	Prophylaxis (IG: 9; CG: 8) On demand (IG: 1; CG: 2)
Moreno‐Segura et al. (2023) [39] RCT	19 IG: 10 CG: 9	IG: 45 ± 8.56 CG: 37.89 ± 13.31	Severe (19)	Prophylaxis (19)
Wilczyński et al. (2023) [45] Case report	1	20	Severe	Prophylaxis
Elnaggar et al. (2024) [35] RCT	48 IG: 24 CG: 24	IG: 13.17 ± 2.2 CG: 12.88 ± 2.52	Moderate (48)	Prophylaxis (48)
Srichumpuang et al. (2024) [44] Non‐RCT	13	20.1 ± 6.8	Moderate (3) Severe (11)	Prophylaxis (13)

*Note*: Sample size was reported as the absolute frequency of participants. Age was expressed in years as mean ± standard deviation or minimum‐maximum age. Haemophilia severity and factor therapy replacement were reported as absolute or relative frequencies for primary studies and as absolute frequencies for systematic reviews.

Abbreviations: AT, aerobic training; CG, control group; CT, combined training; CTG, conventional treatment group; IG, intervention group; N/A, not assessed; P1, participant 1; P2, participant 2; PEMF, pulsed electromagnetic fields; RCT, randomised controlled trial; RT, resistance training; RTPEMF, resistance training combined with pulsed electromagnetic fields.

**TABLE 3:** Overview of the exercise parameters (*n* = 36).

Author (year) Design	Frequency	Intensity	Time (duration)	Type (exercise modalities)
Pelletier et al. (1987) [28] Case report	N/A	2/3 (20%) MVIC	3 weeks	Strengthening (isometric)
Tiktinsky et al. (2002) [20] Prospective and retrospective pilot	3 days/week	RM	48 weeks	Flexibility (stretching) Strengthening (isotonic free weights)
Gomis et al. (2009) [48] Systematic review	N/A		N/A	Aerobic Balance Flexibility Strengthening Proprioceptive
Vallejo et al. (2010) [30] Single‐arm prospective	3 days/week	50%–75% MHR and 2–7 modified Robertson scale	9 weeks	Aerobic (aquatic) Endurance (free active exercise, floaters) Flexibility (stretching) Strengthening (isotonic free active exercise, floaters)
Mulvany et al. (2010) [25] Single arm pre‐test‐post‐test	2 days/week	50%–70% MHR and 40%–75% MVIC	6 weeks	Aerobic (free activity) Flexibility (stretching) Strengthening (isometric, isotonic free weights, stationary resistance equipment, elastic bands, functional activities)
Querol et al. (2011) [50] Systematic review	N/A		N/A	Aerobic Balance Flexibility Strengthening
Souza et al. (2012) [49] Systematic review	2–7 days/week		3–96 weeks	Aerobic Strengthening
Kargarfard et al. (2013) [23] Non‐RCT	3 days/week	50%–74% MHR	8 weeks	Aerobic (aquatic) Flexibility (unspecified) Strengthening (unspecified isotonic)
Eid et al. (2014) [22] RCT	3 days/week	75% Over‐ground speed	12 weeks	Aerobic (treadmill) Endurance (bicycle ergometer, free weights) Flexibility (stretching) Strengthening (isometric)
Parhampour et al. (2014) [26] RCT	3 days/week	50%–60% RM	6 weeks	Strengthening (isotonic free weights, stationary resistance equipment)
Al‐Sharif et al. (2014) [21] RCT	3 days/week	50%–75% MHR	12 weeks	Aerobic (treadmill)
Mohamed & Sherief (2015) [24] RCT	3 days/week	75% Over‐ground speed	12 weeks	Aerobic (bicycle ergometer, treadmill) Balance (unspecified) Flexibility (stretching) Strengthening (isometric)
Cruz et al. (2015) [32] Case report	2 days/week	10 RM and 11–13 RPE (Borg)	87 weeks	Aerobic (bicycle ergometer) Strengthening (isotonic stationary resistance equipment)
Blum (2015) [47] Systematic review	2–3 days/week		4–9 weeks	Aerobic Balance Flexibility Strengthening
Runkel et al. (2016) [29] RCT	2 days/week	10%–70% RPE	36 weeks	Endurance (unspecified) Flexibility (mobility) Strengthening (unspecified resisted isotonic)
Strike et al. (2016) [5] Systematic review	2–5 days/week		4–12 weeks	Aerobic Flexibility Strengthening
Schäfer et al. (2016) [51] Systematic review	1–2 days/week		4–48 weeks	Aerobic Endurance Balance Flexibility Strengthening
Runkel et al. (2017) [43] RCT	2 days/week	10%–70% RPE	36 weeks	Endurance (unspecified) Flexibility (mobility) Strengthening (unspecified resisted isotonic)
Parhampour et al. (2019) [27] RCT	3 days/week	65%–75% MHR and RM	6 weeks	Aerobic (bicycle ergometer, treadmill) Strengthening (isotonic free weights, stationary resistance equipment)
Neelapala et al. (2019) [46] Systematic review	∼3 days/week		3–8 weeks	Aerobic Flexibility Strengthening
Deniz & Güzel (2020) [34] RCT	3 days/week	40%–60% RM	8 weeks	Aerobic (treadmill) Balance (weight‐bearing, swinging anteroposterior‐right and left directions) Flexibility (mobility, stretching) Strengthening (isotonic elastic bands)
Calatayud et al. (2020) [31] RCT	2 days/week	10–20 RM	8 weeks	Strengthening (isotonic elastic bands)
Wagner et al. (2020) [7] Systematic review	2–7 days/week		6–96 weeks	Endurance Balance Flexibility Strengthening
García‐Dasí et al. (2021) [37] Two‐arm prospective	3 days/week	3–4 OMNI‐GSE/RES	16 weeks	Aerobic (walking) Flexibility (mobility, stretching) Strengthening (isotonic elastic bands)
Parhampour et al. (2021) [40] RCT	3 days/week	65%–75% MHR and RM	6 weeks	Aerobic (bicycle ergometer, treadmill) Flexibility (stretching) Strengthening (isotonic free weights, stationary resistance equipment)
Pillard et al. (2021) [42] Exploratory	3 days/week	Maximal exercise test (VO_2_ max, MAP, HR VT‐1 and VT‐2)	6 weeks	Endurance (bicycle ergometer)
Parhampour et al. (2022) [41] RCT	3 days/week	65%–75% MHR and RM	6 weeks	Aerobic (bicycle ergometer, treadmill) Flexibility (stretching) Strengthening (isotonic free weights, stationary resistance equipment)
Gönen et al. (2022) [38] RCT	2 days/week	40%–60% MVIC	12 weeks	Balance (↓ BoS, balance board, eyes closed) Flexibility (mobility) Strengthening (isometric, isotonic body weight, free weights, elastic bands) Proprioceptive (unspecified)
Fares et al. (2022) [36] RCT	3 days/week	50%–60% HRR	8 weeks	Aerobic (bicycle ergometer) Balance (↓ BoS, single‐leg stance) Flexibility (mobility, stretching) Strengthening (unspecified)
Tomschi et al. (2022) [8] Systematic review	1–3 days/week		6–12 weeks	Aerobic
Chimeno‐Hernández et al. (2022) [9] Systematic review	2–7 days/week		4–52 weeks	Aerobic Balance Flexibility Strengthening
Cruz‐Montecinos et al. (2023) [52] RCT	2 days/week	10–20 RM	8 weeks	Strengthening (isotonic elastic bands)
Moreno‐Segura et al. (2023) [39] RCT	3 days/week	3–7 OMNI‐GSE/RES	16 weeks	Aerobic (walking) Flexibility (mobility, stretching) Strengthening (isotonic elastic bands)
Wilczyński et al. (2023) [45] Case report	3 days/week	80% 20 RM	6 weeks	Strengthening (isotonic body weight, free weights)
Elnaggar et al. (2024) [35] RCT	2 days/week	50% MHR and pilot test	12 weeks	Aerobic (bicycle ergometer, treadmill, aquatic) Balance (unspecified) Flexibility (mobility, stretching, plyometric) Strengthening (isometric, isotonic elastic bands) Proprioceptive (leg‐press, multidirectional steps)
Srichumpuang et al. (2024) [44] Non‐RCT	7 days/week	↑ 65% MHR	24 weeks	Aerobic (free activity) Endurance (treadmill, body weight) Balance (postures) Flexibility (mobility, stretching) Strengthening (isotonic body weight)

Abbreviations: BoS, base of support; HR, heart rate; HRR, heart rate reserve; MAP, maximal aerobic power; MHR, maximum heart rate; MVIC, maximum voluntary isometric contraction; N/A, not assessed; RCT, randomised clinical trial; RM, repetition maximum; RPE, rating of perceived exertion; VO_2_ max, maximal oxygen uptake; VT‐1, first ventilatory threshold; VT‐2, second ventilatory threshold.

## 3 Results

### 3.1 Study Selection

**FIGURE 1:**
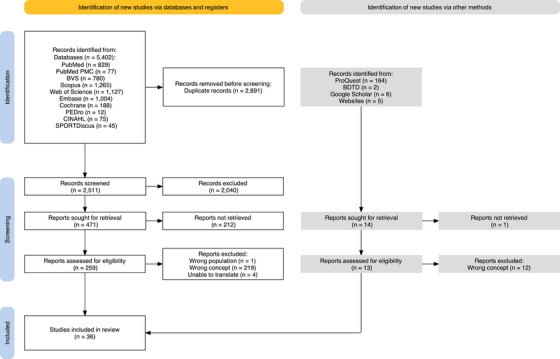
Flowchart of the literature research and the selection process.

The search strategy identified 5.579 references, 5.402 from databases and 177 from grey literature. Following the removal of duplicates and screening of titles and abstracts, 259 references from databases and 13 from grey literature were assessed for eligibility based on full‐text reading. Of these, 224 database references and 12 grey literature references were excluded for not meeting the eligibility criteria, primarily due to wrong concept (219 and 12, respectively) (Appendix S2). The final sample included 36 studies (Figure 1).

### 3.2 Study Characteristics

Of the final sample, 26 (72.2%) were primary evidence sources. Amongst them, 11 (42.3%) studies appeared in more than one source [20, 21, 22, 23, 24, 25, 26, 27, 28, 29, 30], while 15 (57.7%) were unique studies [31, 32, 33, 34, 35, 36, 37, 38, 39, 40, 41, 42, 43, 44, 45]. The unique studies consisted mainly of randomised clinical trials (*n* = 10, 66.7%) [31, 33, 34, 35, 36, 38, 39, 40, 41, 43], followed by case reports (*n* = 2, 13.3%) [32, 45], a non‐randomised clinical trial (*n* = 1, 6.7%) [44], a two‐arm prospective study [37], and an exploratory study (*n* = 1, 6.7%) [42]. The remaining sources were systematic reviews (*n* = 10, 27.8%), which included between 4 [46, 47] and 103 [48] studies (median: 8.5, IQR: 7) [5, 7, 8, 9, 46, 47, 48, 49, 50, 51]. The years of publication ranged from 1987 [28] to 2024 [35, 44]. with the highest number of publications in 2022 (*n* = 5, 13.9%) [8, 9, 36, 38, 41]. Spain had the highest number of publications (*n* = 8, 22.2%) [9, 30, 31, 37, 39, 48, 50, 52], followed by Iran (*n* = 6, 16.7%) [20, 23, 26, 27, 40, 41]. Most studies were conducted in clinical settings (*n* = 28, 77.8%), whereas very few occurred in non‐clinical settings (*n* = 8, 22.2%), such as qualified fitness studios [29, 43], sports facilities [34, 40] or home‐based (Table 1) [37, 39, 42, 44].

### 3.3 Participants

Over half (*n* = 20, 55.6%) of the studies included only adults, 5 (13.9%) included only children [22, 24, 28, 35, 38], and 10 (27.8%) included both [5, 7, 8, 9, 25, 44, 46, 48, 50]. Regarding haemophilia severity, participants had severe (*n* = 9, 25%) [20, 26, 28, 32, 37, 39, 45, 48, 50], moderate (*n* = 8, 22.2%) [21, 22, 23, 27, 35, 40, 41, 46], moderate/severe (*n* = 8, 22.2%) [8, 29, 30, 34, 38, 43, 44, 51], mild/moderate/severe (*n* = 7, 19.4%) [5, 7, 9, 25, 31, 49, 52] or mild/moderate (*n* = 3, 8.3%) haemophilia [24, 36, 42]. Only four (11.1%) studies included participants with inhibitors [29, 30, 43, 45]. Participants were on prophylaxis in 13 (36.1%) studies [26, 27, 32, 35, 36, 37, 38, 39, 40, 41, 44, 45, 51], on demand in three (8.3%) studies [20, 21, 24], and both on prophylaxis and on demand in eight (22.2%) studies [7, 8, 9, 29, 30, 31, 48, 52]. Factor replacement therapy was administered either before or during the intervention in four (11.1%) studies [5, 22, 25, 28], while eight (22.2 %) studies did not specify the treatment regimen [23, 34, 42, 43, 46, 47, 49, 50] (Table 2).

### 3.4 Exercise Parameters

The exercise parameters are outlined in Table 3. Further details on the programmes are provided in Appendix S3.

#### 3.4.1 Frequency

Exercise frequency was set at 3 days per week in over half (*n* = 16, 61.5%) of the primary studies [20, 21, 22, 23, 24, 26, 27, 30, 34, 36, 37, 39, 40, 41, 42, 45]. Eight studies (30.8%) set the frequency at 2 days per week [25, 29, 31, 32, 35, 38, 43, 52], whereas only one study performed daily exercise [44]. In the systematic reviews, the reported exercise frequency ranged from 1 [8, 51] to 2 [5, 7, 9, 47, 49] days per week at minimum (median: 2, IQR: 1) and 2 [51] to 7 [7, 9, 49] days per week at maximum (median: 4, IQR: 4). Two reviews did not report the exercise frequency of the included studies [48, 50].

#### 3.4.2 Intensity

Training intensity in the primary studies was most commonly defined by the maximum number of repetitions (RM) (*n* = 10, 38.5%) [20, 26, 27, 31, 32, 34, 40, 41, 45, 52] and/or maximum heart rate (MHR) (*n* = 9, 34.6%) [21, 23, 27, 30, 35, 36, 40, 41, 44], followed by subjective perceived exertion scales (*n* = 6, 23.1%) [29, 30, 32, 37, 39, 43]. Six (23.1%) studies used different parameters, such as over‐ground speed [22, 24], pilot‐testing [35], maximal exercise test [42] and maximum voluntary isometric contraction (MVIC) [25, 28, 38]. Seventy percent (*n* = 7) of the reviews did not report on how intensity was defined [5, 7, 9, 47, 48, 50, 51].

#### 3.4.3 Time (Duration)

Exercise interventions in primary studies ranged from 3 [28] to 87 [32] weeks (median: 8.5, IQR: 9). Most lasted for 6 weeks (*n* = 7, 26.9%) [25, 26, 27, 40, 41, 42, 45], followed by eight (*n* = 5, 19.2%) [23, 31, 34, 36, 52] and 12 weeks (*n* = 5, 19.2%) [21, 22, 24, 35, 38]. Systematic reviews reported a minimum duration of 3 [46, 49] to 6 weeks [7, 8] (median: 4, IQR: 1.5), and a maximum of 8 [46] to 96 [7, 49] weeks (median: 30, IQR: 63.5). Two reviews did not report the on how long the interventions lasted [48, 50].

#### 3.4.4 Type (Modalities)

Interventions consisted of land‐based exercises (*n* = 24, 66.7%), water‐based exercises (*n* = 4, 11.1%) [23, 30, 46, 47] or a combination of both (*n* = 8, 22.2%) [5, 7, 8, 35, 48, 49, 50, 51], categorised into six exercise modalities: strengthening, flexibility, aerobic, balance, endurance and proprioceptive training.

Strengthening exercises were conducted in nearly all primary studies (*n* = 24, 92.3%), except for two studies [21, 42]. The interventions consisted of isotonic exercises (*n* = 17, 65.4%) [20, 23, 26, 27, 29, 30, 31, 32, 34, 37, 39, 40, 41, 43, 44, 45], isometric exercises (*n* = 3, 11.5%) [22, 24, 28] or a combination of both (*n* = 2, 8.3%) [25, 35, 38]. One (6.3%) study combined isometric, isotonic and plyometric exercises [35]. Free weights [20, 25, 26, 27, 38, 40, 41, 45] and elastic bands [25, 31, 34, 35, 37, 38, 39, 52] were the most commonly used forms of resistance (*n* = 8, 40%), followed by stationary resistance equipment (*n* = 6, 30%) [25, 26, 27, 32, 40, 41] and body weight (*n* = 3, 15%) [38, 44, 45]. Other types of resistance included floaters [30] and functional activities, such as walking, standing up from a seated position, and climbing stairs [25]. Four (16.7%) studies did not report the type of resistance used [23, 29, 36, 43], and one did not specify the type of strengthening exercise [36].

Flexibility (*n* = 17, 65.4%) and aerobic (*n* = 16, 61.5%) exercises were conducted in over half of the primary studies. Flexibility interventions involved static stretching (*n* = 13, 81.3%) [20, 22, 24, 25, 30, 34, 35, 36, 37, 39, 40, 41, 44], and mobility (*n* = 9, 56.3%) [29, 34, 35, 36, 37, 38, 39, 43, 44]. Six (37.5%) studies combined stretching and mobility exercises [34, 35, 36, 37, 39, 44]. One study did not report the type of flexibility exercises used [23]. Aerobic interventions included treadmill (*n* = 8, 57.1%) [21, 22, 24, 27, 34, 35, 40, 41], bicycle ergometer (*n* = 7, 50%) [21, 22, 24, 27, 34, 35, 40, 41], water‐based exercise (*n* = 3, 21.4%) [23, 30, 35] and walking (*n* = 2, 14.3%) [37, 39]. Five (35.7%) studies combined treadmill and bicycle ergometer interventions [24, 27, 35, 40, 41], one of which also included water‐based exercises [35]. Two studies allowed participants to choose the type of aerobic exercise [44] or did not report the type of aerobic exercise used [25]. Balance (*n* = 6, 23.1%) [24, 34, 35, 36, 38, 44], endurance (*n* = 6, 23.1%) [22, 29, 30, 42, 43, 44] and proprioceptive (*n* = 2, 7.7%) [35, 38] training were the least commonly used modalities.

Of the primary studies, seven (26.9%) conducted unimodal exercise programmes with strengthening (*n* = 5, 19.2%) [26, 28, 31, 45, 52], aerobic (*n* = 1, 3.9%) [21] or endurance (*n* = 1, 3.9%) [42] interventions. The remaining studies (*n* = 19, 73.1%) conducted multimodal exercise programmes, incorporating two [20, 27, 32] to five [35, 44] modalities per programme. Most multimodal programmes combined three exercise modalities (*n* = 8, 30.8%) [23, 25, 29, 37, 39, 40, 41, 43], followed by four modalities (*n* = 6, 23.1%) [22, 24, 30, 34, 36, 38]. The most common combination was strengthening, flexibility and aerobic exercises (*n* = 6, 23.1%) [23, 25, 37, 39, 40, 41], followed by balance training combined with the previous modalities (*n* = 3, 11.5%) [24, 34, 36].

Strengthening and aerobic exercises were reported in nearly all (*n* = 9, 90%) but two systematic reviews, respectively [7, 8]. Over half of the reviews also reported flexibility (*n* = 8, 80%) [5, 7, 9, 46, 47, 48, 50, 51] and balance (*n* = 6, 60%) [7, 9, 47, 48, 50, 51] exercises. Endurance [7, 51] and proprioceptive [48] training were the least reported modalities.

### 3.5 Tendencies in Exercise Prescription

**FIGURE 2:**
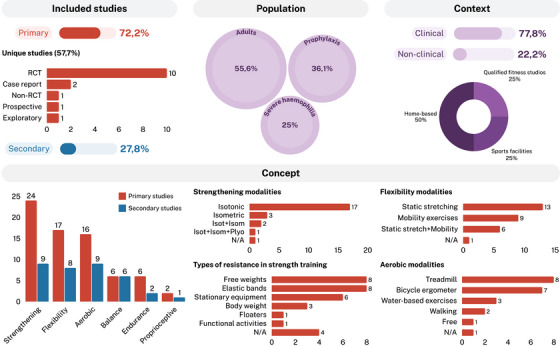
Visual summary of the review's findings (*n* = 36). Isom, isometric; Isot, isotonic; N/A, not assessed; Plyo, plyometric.

Figure 2 shows a visual summary of the review's findings according to the ‘PCC’ mnemonic. Table 4 summarises the patterns, advances and gaps found in the literature, as well as the evidence for practice and research recommendations [53].

**TABLE 4:** PAGER framework of exercise programme patterns in people with haemophilia [53].

Pattern (P)	Advances (A)	Gaps (G)	Evidence for practice (E)	Research recommendations (R)
Progressive strength training appears to be associated with improvements in muscle strength [20, 22, 25, 26, 28, 29, 31, 32, 35, 41, 44] and other health outcomes, including function [22, 25, 26, 27, 31, 32, 33, 35, 39, 45], pain [20, 26, 31, 37, 39] and QoL [34, 37, 43, 44, 45].	Light‐to‐moderate intensity (50%–60% RM; Borg 11–13) seems effective for PwH [26, 29]. Moderate‐to‐vigorous intensity (60%–70% RM) may be applied without inducing acute bleeding depending on the clinical/bleeding and functional profile of the patient, with adequate prophylaxis and gradual progression throughout the training protocol [27, 31, 33, 40, 41, 44].	Protocol heterogeneity and lack of standardisation in defining intensity. Few long‐term follow‐ups and under‐representation of people with inhibitors.	Prescription tends to 6–12 weeks, 30–90 min/session, 2–3×/week, starting at light‐to‐moderate intensity (50%–60% RM) with gradual progression (absence of adverse events and adequate factor coverage). Elastic bands are accessible and recommended for home training. Closed kinetic chain lower‐limb exercises in children appear to improve proprioception and PA levels [38].	Multicentre RCTs comparing intensities and resistance levels according to haemophilia severity. Dose‐response and qualitative studies on home adherence.
Aerobic and endurance training appear to be associated with improvements in physical fitness/pulmonary function [30, 36, 42], function [22, 25, 29, 30, 35, 39], strength [21, 22, 23, 29, 35, 41, 44], QoL [34, 37, 43, 44] and balance [24, 29].	Light‐to‐moderate intensity (50%–75% MHR) appears safe and beneficial for most PwH [23, 25, 27, 29, 30, 35, 36, 40, 41, 43].	Lack of comparisons between aerobic exercise types and absence of long‐term follow‐up. Under‐representation of people with inhibitors.	Prescription tends to 6–12 weeks, 20–60 min/session, 2–3×/week, light‐to‐moderate intensity (50%–75% MHR; Borg 11–13), with individualisation and progression according to tolerance (no adverse events and adequate factor coverage). Treadmill training in children appears to yield better results than cycle ergometer, especially for balance and gait [24].	RCTs comparing aquatic, land‐based and interval training, with long‐term follow‐up to assess maintenance of gains.
Combined training (resistance + aerobic) appears to be associated with improvements in bone [22], metabolic [27, 40] and inflammatory markers [27] in adults with moderate haemophilia.	Moderate‐to‐vigorous intensity (65%–75% RM and MHR), with biweekly progression over six weeks, appears feasible in adults with moderate haemophilia and overweight under FVIII prophylaxis, without acute bleeding [27, 40, 41].	Short‐duration studies with small samples focusing on moderate haemophilia. Unknown impact in severe cases or those with inhibitors.	Prescription tends to 6 weeks, 40–45 min/session, 3×/week, moderate‐to‐vigorous intensity (65%–75% RM + 65%–75% MHR).	RCTs ≥12 weeks comparing combined versus isolated training, with long‐term follow‐up. Studies in severe haemophilia and people with inhibitors.
Flexibility training appeared in more than half of the primary studies as part of multimodal programmes, primarily in the form of static stretching or a combination of static stretching and mobility exercises.	Suggests recognition of flexibility as a key component of musculoskeletal health in PwH, essential for maintaining joint mobility and preventing contractures.	Included studies did not report ROM as the primary outcome, except for one RCT [31]. Lack of studies evaluating flexibility training as an isolated modality in PwH, and lack of standardisation regarding the type of exercise (static stretching vs. mobility) and holding time.	Prescription tends to encompass flexibility alongside strengthening and aerobic exercises as part of multimodal fitness programmes, 2–3×/week, 10–30 seconds per stretch within a comfortable range, as recommended by the ACSM.	RCTs isolating the effects of flexibility exercises and comparative studies evaluating frequency and stretch duration on joint mobility, pain, and function in PwH.
Neuromotor training was the least used modality. Only 23.1% of studies included balance exercises [24, 34, 35, 36, 38, 44] and 7.7% included proprioceptive exercises [35, 38].	Suggests a rising awareness of the neuromotor component's importance in joint stability for PwH over the past decade.	Scarce and poorly detailed neuromotor programmes. Uncertainty about frequency, duration and integration with other modalities.	Prescription tends to be integrated into multimodal programmes, starting on stable surfaces and increasing complexity (reduced BoS, eyes closed, unstable surfaces etc.) according to progression.	RCTs testing isolated neuromotor training protocols.
Most interventions were multimodal, combining strengthening, flexibility and aerobic exercises [23, 25, 37, 39, 40, 41].	Suggests a trend aligned with ACSM recommendations.	Few comparisons between multimodal and unimodal effects. Additive benefits and time allocated to each modality remain unclear.	Combining resistance, flexibility and aerobic exercises appears feasible and safe for PwH. Typical prescription: 6–12 weeks, 2–3×/week.	RCTs isolating the effects of each component and testing different sequencing within the same programme.
Intervention safety appears to be linked to the inclusion of participants primarily under prophylaxis with clotting factor concentrates, but treatment reporting is inconsistent.	Suggests that prophylaxis or factor replacement before exercise sessions enhances feasibility and safety across haemophilia severities.	Incomplete reporting on type of prophylaxis, dosage and timing limits generalisation, especially in resource‐limited settings.	Current evidence mostly reflects individuals on prophylaxis, suggesting cautious prescription for those on on‐demand therapy, considering factor availability and timing.	Future studies should detail treatment regimens, administration timing and bleeding outcomes.
Considerable heterogeneity exists in joint status and treatment regimens among studies, limiting the formulation of universal recommendations.	Highlights the need to tailor exercise according to joint status and therapeutic regimen (factor‐based or non‐factor therapies, prophylaxis or on‐demand).	Lack of studies on exercise dosage in PwH using non‐factor therapies.	Exercise prescription for PwH should consider joint status, treatment regimen and therapeutic access. Safe and effective strategies in high‐resource or novel therapy settings may not be generalisable to resource‐limited contexts.	RCTs testing different exercise modalities among PwH on non‐factor therapies.

Abbreviations: ACSM, American College of Sports Medicine; BoS, base of support; FVIII, factor VIII; MHR, maximum heart rate; PwH, people with haemophilia; QoL, quality of life; RCT, randomised clinical trial; RM, repetition maximum.

## 4 Discussion

This is the first comprehensive scoping review mapping exercise programme parameters for PwH across various disease severities. A total of 36 sources were included, mainly randomised clinical trials and systematic reviews, with a few quasi‐experimental studies. Of the primary sources included, 15 were unique studies. The exercise programmes varied reasonably; each parameter will be discussed individually in the following paragraphs.

### 4.1 Frequency

Exercise frequency in the primary studies was mainly set at 3 days per week, followed by 2 days per week; similarly, the systematic reviews reported a median frequency for 2–4 days per week. The American College of Sports Medicine (ACSM) guidelines for exercise testing and prescription recommend aerobic training 3–5 times per week, resistance training 2–3 times and flexibility exercises 2–3 times [4, 54]. Blamey et al. published a review article exploring elements of physiotherapy exercise programmes for haemophilia, recommending resistance training 2–4 times per week to achieve significant strength gains based on the findings of previous studies in untrained individuals [55]. The authors suggested performing flexibility training daily or at least 2–3 times weekly based on the evidence of significant gains in ROM from a systematic review [55]. As seen in the included studies, the exercise programmes were mostly aligned with the aforementioned recommendations. Considering that there is no definitive optimal exercise frequency for adaptation to occur, an individualised approach is recommended, with training frequency tailored to account for each person's physical function, health status and goals [4, 56].

### 4.2 Intensity

Training intensity in the primary studies was mostly defined by the RM or MHR, followed by perceived exertion. The ACSM's guidelines recommend moderate (64%–76% MHR) to vigorous (77%–95% MHR) intensity for aerobic exercise in most adults, with mild (57%–63% MHR) to moderate intensity potentially beneficial for deconditioned individuals; for resistance training, it is recommended moderate to high intensity (60%–70% of RM) for beginners, and very mild to mild intensity (40%–50% of RM) for previously sedentary individuals [4, 54].

Randomised controlled trials (RCTs) suggest that engaging in a 45‐min exercise programme including progressive resistance and aerobic training at moderate‐to‐vigorous intensity thrice‐a‐week over 6 weeks can significantly improve the lipid profile and quality of life of overweight adults with moderate haemophilia without inducing bleeding episodes [40], and that both combined and resistance training are safe, effective non‐pharmacological strategies for enhancing muscle strength and preventing musculoskeletal complications [41]. Additionally, an 8‐week RCT of an aerobic exercise programme using a cycle ergometer with a target heart rate reserve (HRR) of 50%–60% found that moderate‐intensity training significantly improved lung function in adults with moderate haemophilia on prophylactic treatment [36]. Consistent with the findings above, a recent systematic review of aerobic exercise in PwH indicates that training intensity can be safely performed at approximately 55%–75% of MHR, depending on the type of aerobic exercise and the level of joint impairment. The authors suggest using perceived exertion instruments, such as the Borg scale, to define training intensity, as severe joint impairments could prevent reaching the predefined heart rates [8].

Another systematic review suggests that resistance training intensities lower than those typically required to increase strength in healthy individuals seem to be effective for PwH, as observed in a two‐arm prospective study with severely haemophilic participants [7, 37]. The authors found that a 16‐week home‐based exercise programme including low‐intensity aerobic and strengthening exercises, combined with cognitive‐behavioural therapy sessions, significantly improved chronic pain self‐efficacy, quality of life and emotional status, while reducing pain and kinesiophobia.

While moderate‐to‐vigorous‐intensity is recommended for most adults, PwH, especially those with joint impairments, should have intensity tailored to individual tolerance levels, possibly with greater emphasis on low‐impact exercises. The importance of individualised exercise prescription is highlighted by a case report of a 20‐year‐old athlete with severe haemophilia A and an inhibitor. While on a new prophylactic drug, he completed a 6‐week, thrice‐weekly intensive strength training programme targeting limb and trunk muscles at 80% of the maximum load, calculated using the 20‐rep method. The authors observed improvements in quality of life and lower limb strength, with no musculoskeletal injuries or bleeding episodes during the intervention or 3‐month follow‐up [45]. Evidently, these findings should not be generalised to other PwH.

### 4.3 Time (Duration)

Exercise interventions in primary studies mostly lasted for 6 weeks, followed by 8 and 12 weeks, whereas the systematic reviews reported a median duration of 4–30 weeks. Authors suggest that a resistance training duration of 6 weeks can significantly improve strength in PwH, while 2 weeks of training did not yield significantly strength gains [7]. Likewise, Blamey et al. recommend approximately 6–8 weeks of resistance training to achieve significant improvements [55]. In a non‐randomised clinical trial, individualised exercise protocols were implemented over a 6‐month period for participants of various age groups and joint conditions, all of whom had moderate and severe haemophilia and were on low‐dose prophylaxis. The programmes targeted moderate‐to‐vigorous‐intensity training, including freestyle aerobic activity three times a week, with weekly sessions of bodyweight strength training, endurance, flexibility and balance exercises. The results indicate that this tailored approach improved bleeding prevention, musculoskeletal status and quality of life while reducing FVIII consumption, which could potentially optimise haemophilia care in resource‐limited settings [44]. Additional research is needed to define the optimal duration for PwH in other exercise modalities. However, the length of the programme should still reflect the individual's responses and goals.

### 4.4 Type (Modalities)

Strengthening exercises were conducted in nearly all primary studies and reported in almost all systematic reviews, primarily isotonic exercises. Free weights and elastic bands were the most used types of resistance. In PwH, particularly adults, muscle strength is significantly lower than in healthy individuals; therefore, strength training is of utmost importance [48, 57, 58]. A randomised clinical trial found that an 8‐week, twice‐weekly progressive exercise programme incorporating moderate‐to‐vigorous‐intensity elastic resistance training targeting knee, elbow and ankle joints muscles improved the quality of life dimension of joint damage perception, and functional abilities in adults with haemophilia, without causing exercise‐induced bleeding episodes [52]. Both elastic bands and free weights are frequently used and recommended in the literature for providing resistance in strength training for PwH. Due to their portability and affordability, Wagner et al. suggest that elastic bands are the best option for home‐based training [7].

Flexibility exercises, primarily involving stretching or a combination of stretching and mobility exercises, were conducted in over half of the primary studies and reported in over half of the systematic reviews. None of the unique included studies conducted interventions that consisted solely of flexibility exercises, and those that included them as part of a multimodal programme did not report ROM as a main outcome, with the exception of one RCT [31].

Maintaining an adequate ROM in all joints is essential for optimal musculoskeletal function. This is particularly important for PwH, as joint mobility is crucial for preventing articular and muscular contractures [6, 55]. Although stretching exercises are recommended to increase flexibility and ROM, there are no standard guidelines for their prescription—even for healthy individuals [55]. In this context, it is challenging to interpret the available evidence and apply it to clinical practice in PwH. The effectiveness of flexibility‐based interventions may be limited by the irreversible joint remodelling that can occur in PwH, which is associated with a decrease in joint ROM as arthropathy progresses [59]. It is generally recommended that fitness programmes for PwH include stretching, with particular emphasis on static stretching [55]. In healthy adults, joint ROM can be improved after engaging in regular stretching 2–3 times per week for approximately 3–4 weeks [54]. The ACSM's guidelines recommend that flexibility exercises be tailored to the individuals’ level of discomfort and within their current ROM, with each stretch sustained for 10–30 s to maximise effectiveness; this approach is well‐aligned with the recommendations for PwH [4, 55].

Aerobic exercises, primarily a combination of treadmill and bicycle ergometer, were also conducted in over half of the primary studies and reported in nearly all systematic reviews. Tomschi et al. advise not using a bicycle ergometer in PwH with severe restrictions in knee ROM, as they may experience difficulties in movement or pain; treadmill exercise can be recommended if the lower limbs are not affected [8].

The least commonly used exercise modalities in primary studies were balance, endurance and proprioceptive training; similarly, endurance and proprioceptive training were the least reported modalities in systematic reviews. In a 6‐week exploratory study, adults with mild to moderate haemophilia engaged in a home‐based personalised endurance training programme. Each 45‐min session included interval training on a cycle ergometer, alternating high‐intensity periods based on the performance achieved at the second ventilatory threshold with recovery periods at moderate intensity. The programme significantly improved endurance power without bleeding events or functional limitations, and slightly increased FVII levels compared to rest in participants with mild haemophilia, possibly due to the personalisation and standardisation of the exercise dose [42].

The ACSM's guidelines recommend neuromotor exercises, including balance and proprioceptive training, as essential components of a comprehensive exercise programme for older adults; however, the guidelines do not provide specific recommendations for young and middle‐aged adults [54]. PwH, particularly those with haemophilic arthropathy (HA), may experience deficits in proprioception akin to those seen in patients with osteoarthritis that are further affected by repeated joint bleeds, contributing to alterations in postural balance and an increased risk of injury [6, 55]. Consequently, the musculoskeletal complications associated with HA elevate the fall risk among PwH, with loss of balance frequently identified as the primary contributing factor to falls in this population [60]. Given that falls can precipitate severe outcomes, including fractures, chronic pain, diminished mobility and functional decline, fall prevention constitutes a vital aspect of comprehensive healthcare for PwH, achievable through neuromotor training. Negrier et al. recommend incorporating balance and coordination training to enhance proprioception and, subsequently, reduce the risk of trauma [2].

A 12‐week RCT investigated the effects of a twice‐weekly structured programme combining close kinetic chain (CKC) strengthening and balance exercises. Compared with conventional programmes reported in the literature, CKC exercises improved proprioception and PA levels in paediatric patients with moderate to severe haemophilia and at least one target knee joint [38]. Thus, exercise programmes for PwH should strive to include neuromotor training modalities in multimodal programmes, starting on stable surfaces and gradually progressing to more complex tasks according to functional adaptation and clinical safety [2, 9].

### 4.5 Tendencies in Exercise Prescription

A review of the literature revealed relevant patterns among the parameters of exercise programmes for PwH, which may broadly guide the prescription of exercise programmes for this population. Progressive strength and aerobic training, often integrated into multimodal programmes, emerge as common and potentially beneficial, particularly when performed under supervision and adequate factor coverage—preferably prophylaxis—with individualised parameters.

Flexibility training was also widely reported in association with other modalities, although it still lacks investigation of its isolated effects and protocol standardisation, both in healthy individuals and in PwH. Furthermore, neuromotor exercises remain underutilised despite their importance for joint stability and the prevention of falls and trauma, particularly in individuals with HA.

A consistent trend across studies indicates a preference for supervised, multimodal programmes involving light to moderate intensity exercise, performed 2–3 times a week for 6–12 weeks without the occurrence of adverse events, suggesting the feasibility and tolerability of such parameters. However, the heterogeneity of protocols and samples among studies, particularly regarding joint status and haemophilia treatment regimens, limits the generalisability of the evidence.

These findings should therefore be interpreted with caution, as the scoping review methodology does not include a critical appraisal of study quality or risk of bias, and thus cannot determine the efficacy or safety of the interventions. Clinical application should prioritise prescriptions tailored to joint status, bleeding phenotype, therapeutic regimen and healthcare access conditions. An individualised and closely monitored approach is recommended, prioritising low‐impact exercises and progressive adjustments in intensity, frequency and/or duration according to each individual's functional capacity and response. These findings may support safer and more personalised exercise prescriptions while reinforcing the need for controlled, long‐term studies to determine the optimal and safe exercise dosage for PwH across different disease severities and therapeutic contexts.

### 4.6 Limitations of the Available Evidence

In the unique primary studies, the main limitation reported was the small sample sizes [31, 33, 37, 38, 39, 40, 41, 44]. Other limitations included: absence of long‐term follow‐up [34, 35, 37, 39, 40], absence of participant or assessor blinding [34, 35, 38, 40], subjective outcome assessments [33, 34, 37], absence of separate analysis of intervention components to determine their individual contributions to the outcomes [37, 39], and absence of control over external factors that could potentially influence PA performance [40, 45].

Likewise, authors of the included systematic reviews note a scarcity of research on exercise in PwH, with the existing literature often exhibiting methodological drawbacks and low evidence levels, contributing to small sample sizes in systematic reviews, and the inclusion of non‐randomised or non‐controlled trials, thereby reducing the confidence in the findings [5, 7, 8, 9]. Additionally, the heterogeneity of interventions, sample sizes and participant characteristics across studies prevents the results from being pooled in a meta‐analysis, which could provide a definitive recommendation on the optimal dosage for this population [5].

### 4.7 Review Limitations

There are some limitations to this scoping review. Authors of publications that could not be retrieved in full text were not contacted, and four studies that potentially met the inclusion criteria were excluded from the review because they could not be freely translated into English/Portuguese. Additionally, sources reporting exercise programmes paired with other physiotherapy interventions were excluded when it was not possible to extract the exercise parameters individually. As a result, some potentially relevant publications may have been omitted. Furthermore, the inclusion of both systematic reviews and primary studies may have introduced the possibility of double‐counting, which could have led to unintended duplication within the mapped evidence. Finally, because the search strategy was limited to a specific set of databases and sources, it is possible that relevant publications were not included. A wider search of information sources could have resulted in more publications being included.

## 5 Conclusion

The findings of the included studies suggest that exercise programmes are consistent with the existing evidence for PwH, and that individualised approaches to exercise prescription are essential. The predominance of strengthening, flexibility and aerobic exercises was to be expected, as these modalities are the primary focus of PA guidelines for healthy individuals; thus, it is logical that studies would seek to adapt these recommendations to PwH. However, it is important to acknowledge the limitations of this review, including the lack of contact with authors of publications that could not be retrieved in full text, the exclusion of studies due to language barriers or due to the exercise programmes reported being paired with other physiotherapy interventions. Future research should focus on developing high‐quality, evidence‐based, standardised protocols to establish clear guidelines for exercise prescription in PwH, and to incorporate neuromotor training to address proprioceptive deficits and reduce injury risk.

## Author Contributions

*Study design*: Maísa P. A. Veríssimo, Flávia de O. M. Maia, Renata C. Gasparino. *Literature search*: Maísa P. A. Veríssimo. *Data collection*: Maísa P. A. Veríssimo, Tatiana N. Kuhn, Renata C. Gasparino. *Data analysis and interpretation*: Maísa P. A. Veríssimo, Tatiana N. Kuhn, Renata C. Gasparino. *Discussion of results*: Maísa P. A. Veríssimo, Renata C. Gasparino. *Writing and/or critical revision of the content*: Maísa P. A. Veríssimo, Janaína B. S. Ricciardi, Carolina Kosour, Mônica P. A. Veríssimo, Flávia de O. M. Maia, Olga M. P. L. Ribeiro, Renata C. Gasparino. *Review and final approval of the final version*: Maísa P. A. Veríssimo, Tatiana N. Kuhn, Janaína B. S. Ricciardi, Carolina Kosour, Mônica P. A. Veríssimo, Flávia de O. M. Maia, Olga M. P. L. Ribeiro, Renata C. Gasparino.

## Funding

This study was financed by the Coordenação de Aperfeiçoamento de Pessoal de Nível Superior—Brasil (CAPES)—Finance Code 001.

## Ethics Statement

This scoping review involved no new research with humans or animals and uses only data from previously published studies; ethical approval, consent and regulatory compliance therefore rest with the original sources.

## Conflicts of Interest

Janaína B. S. Ricciardi has received reimbursement for attending a congress from Novo Nordisk, Roche, Sanofi and Takeda, and has received fees for speaking and/or consulting from Novo Nordisk, Roche, Sanofi and Takeda. Mônica P. A. Veríssimo has received reimbursement for attending a congress from Novo Nordisk and Takeda, and has received a fee for speaking from Novo Nordisk. None of the other authors have any conflicts of interest to disclose concerning this review. A version of this paper was presented by Maísa P. A. Veríssimo at the Congress of Physiotherapy and Occupational Therapy of the State of São Paulo (CONFITOSP24), held on November 30th and December 1st, 2024, in the city of São Paulo, São Paulo, Brazil.

## Supporting information




**Supporting File 1**: hae70225‐sup‐0001‐Appendices.docx

## Data Availability

The data that support the findings of this study are available from the corresponding author upon reasonable request.
