# Supplementary material for: Exercise Programmes for People With Haemophilia: A Scoping Review
Source: Haemophilia. 2026 Feb 20;32(2):381–401. doi: 10.1111/hae.70225 (PMC12984475; doi:10.1111/hae.70225)
Supplement: Supplementary file 1 — Supporting File 1: hae70225‐sup‐0001‐Appendices.docx [file HAE-32-381-s001.docx]

# Appendix 1 - Full search strategy

| Source | Search strategy | N |
| --- | --- | --- |
| PubMed | (((((((((Hemophilia A[MeSH Terms]) OR ("Hemophilia A"[Title/Abstract] OR "Hemophilia As"[Title/Abstract] OR Hemophilia[Title/Abstract] OR "Congenital Hemophilia A"[Title/Abstract] OR "Classic Hemophilia"[Title/Abstract] OR Haemophilia[Title/Abstract] OR "Autosomal Hemophilia A"[Title/Abstract] OR "Factor VIII Deficiency"[Title/Abstract])) OR ("ahf deficiency"[Title/Abstract] OR "ahg deficiency"[Title/Abstract] OR "classic hemophilia"[Title/Abstract] OR "factor viii deficiency"[Title/Abstract] OR "haemophilia a"[Title/Abstract] OR "hemophilia plasma"[Title/Abstract] OR "true haemophilia"[Title/Abstract] OR "true hemophilia"[Title/Abstract])) OR ((Hemophilia B[MeSH Terms]) OR ("Hemophilia B"[Title/Abstract] OR "Factor IX Deficiencies"[Title/Abstract] OR "Factor IX Deficiency"[Title/Abstract] OR "Hemophilia B Leyden"[Title/Abstract] OR "Hemophilia B(M) "[Title/Abstract] OR "Plasma Thromboplastin Component Deficiency"[Title/Abstract] OR "F9 Deficiency"[Title/Abstract] OR "Christmas Disease"[Title/Abstract] OR "Haemophilia B"[Title/Abstract]))) OR ("christmas disease"[Title/Abstract])) OR ("haemophilia (PWH) "[Title/Abstract])) OR (haemophilia[Title/Abstract])) OR (PWH[Title/Abstract])) OR ("people with haemophilia"[Title/Abstract])) AND ((("Physical Therapy"[Title/Abstract]) OR ((Exercise[MeSH Terms]) OR (Exercise[Title/Abstract] OR Exercises[Title/Abstract] OR "Physical Activity"[Title/Abstract] OR "Physical Activities"[Title/Abstract] OR "Physical Exercise"[Title/Abstract] OR "Physical Exercises"[Title/Abstract] OR "Acute Exercise"[Title/Abstract] OR "Acute Exercises"[Title/Abstract] OR "Isometric Exercises"[Title/Abstract] OR "Isometric Exercise"[Title/Abstract] OR "Aerobic Exercise"[Title/Abstract] OR "Aerobic Exercises"[Title/Abstract] OR "Exercise Training"[Title/Abstract] OR "Exercise Trainings"[Title/Abstract]))) OR ((((Exercise Therapy[MeSH Terms]) OR ("Exercise Therapy"[Title/Abstract] OR "Remedial Exercise"[Title/Abstract] OR "Remedial Exercises"[Title/Abstract] OR "Exercise Therapies"[Title/Abstract] OR "Rehabilitation Exercise"[Title/Abstract] OR "Rehabilitation Exercises"[Title/Abstract])) OR ("corrective exercise"[Title/Abstract] OR "exercise movement techniques"[Title/Abstract] OR "exercise treatment"[Title/Abstract] OR "kinesitherapeutic exercises"[Title/Abstract] OR "kinesitherapeutic methodology"[Title/Abstract] OR "kinesitherapeutic treatment"[Title/Abstract] OR kinesitherapy[Title/Abstract] OR "specialized kinesitherapeutic methodology"[Title/Abstract] OR kinesiotherapy[Title/Abstract])) OR (("physical therapy techniques"[Title/Abstract] OR "physical treatment"[Title/Abstract] OR "physio therapy"[Title/Abstract] OR physiotherapy[Title/Abstract]) OR ((Physical Therapy Modalities[MeSH Terms]) OR ("Physical Therapy Modalities"[Title/Abstract] OR "Physical Therapy Modality"[Title/Abstract] OR "Physiotherapy (Techniques) "[Title/Abstract] OR "Physical Therapy Techniques"[Title/Abstract] OR "Physical Therapy Technique"[Title/Abstract] OR "Group Physiotherapy"[Title/Abstract] OR "Physical Therapy"[Title/Abstract] OR "Neurological Physiotherapy"[Title/Abstract] OR Neurophysiotherapy[Title/Abstract]))))) | 829 |
| PubMed PMC | (((((((((Hemophilia A[MeSH Terms]) OR ("Hemophilia A"[Title/Abstract] OR "Hemophilia As"[Title/Abstract] OR Hemophilia[Title/Abstract] OR "Congenital Hemophilia A"[Title/Abstract] OR "Classic Hemophilia"[Title/Abstract] OR Haemophilia[Title/Abstract] OR "Autosomal Hemophilia A"[Title/Abstract] OR "Factor VIII Deficiency"[Title/Abstract])) OR ("ahf deficiency"[Title/Abstract] OR "ahg deficiency"[Title/Abstract] OR "classic hemophilia"[Title/Abstract] OR "factor viii deficiency"[Title/Abstract] OR "haemophilia a"[Title/Abstract] OR "hemophilia plasma"[Title/Abstract] OR "true haemophilia"[Title/Abstract] OR "true hemophilia"[Title/Abstract])) OR ((Hemophilia B[MeSH Terms]) OR ("Hemophilia B"[Title/Abstract] OR "Factor IX Deficiencies"[Title/Abstract] OR "Factor IX Deficiency"[Title/Abstract] OR "Hemophilia B Leyden"[Title/Abstract] OR "Hemophilia B(M) "[Title/Abstract] OR "Plasma Thromboplastin Component Deficiency"[Title/Abstract] OR "F9 Deficiency"[Title/Abstract] OR "Christmas Disease"[Title/Abstract] OR "Haemophilia B"[Title/Abstract]))) OR ("christmas disease"[Title/Abstract])) OR ("haemophilia (PWH) "[Title/Abstract])) OR (haemophilia[Title/Abstract])) OR (PWH[Title/Abstract])) OR ("people with haemophilia"[Title/Abstract])) AND ((("Physical Therapy"[Title/Abstract]) OR ((Exercise[MeSH Terms]) OR (Exercise[Title/Abstract] OR Exercises[Title/Abstract] OR "Physical Activity"[Title/Abstract] OR "Physical Activities"[Title/Abstract] OR "Physical Exercise"[Title/Abstract] OR "Physical Exercises"[Title/Abstract] OR "Acute Exercise"[Title/Abstract] OR "Acute Exercises"[Title/Abstract] OR "Isometric Exercises"[Title/Abstract] OR "Isometric Exercise"[Title/Abstract] OR "Aerobic Exercise"[Title/Abstract] OR "Aerobic Exercises"[Title/Abstract] OR "Exercise Training"[Title/Abstract] OR "Exercise Trainings"[Title/Abstract]))) OR ((((Exercise Therapy[MeSH Terms]) OR ("Exercise Therapy"[Title/Abstract] OR "Remedial Exercise"[Title/Abstract] OR "Remedial Exercises"[Title/Abstract] OR "Exercise Therapies"[Title/Abstract] OR "Rehabilitation Exercise"[Title/Abstract] OR "Rehabilitation Exercises"[Title/Abstract])) OR ("corrective exercise"[Title/Abstract] OR "exercise movement techniques"[Title/Abstract] OR "exercise treatment"[Title/Abstract] OR "kinesitherapeutic exercises"[Title/Abstract] OR "kinesitherapeutic methodology"[Title/Abstract] OR "kinesitherapeutic treatment"[Title/Abstract] OR kinesitherapy[Title/Abstract] OR "specialized kinesitherapeutic methodology"[Title/Abstract] OR kinesiotherapy[Title/Abstract])) OR (("physical therapy techniques"[Title/Abstract] OR "physical treatment"[Title/Abstract] OR "physio therapy"[Title/Abstract] OR physiotherapy[Title/Abstract]) OR ((Physical Therapy Modalities[MeSH Terms]) OR ("Physical Therapy Modalities"[Title/Abstract] OR "Physical Therapy Modality"[Title/Abstract] OR "Physiotherapy (Techniques) "[Title/Abstract] OR "Physical Therapy Techniques"[Title/Abstract] OR "Physical Therapy Technique"[Title/Abstract] OR "Group Physiotherapy"[Title/Abstract] OR "Physical Therapy"[Title/Abstract] OR "Neurological Physiotherapy"[Title/Abstract] OR Neurophysiotherapy[Title/Abstract]))))) | 77 |
| BVS / BIREME  MEDLINE (748)  LILACS (20)  WPRIM (9)  IBECS (3)  CUMED (1) | (("Hemophilia A" OR "Hemophilia As" OR hemophilia OR "Congenital Hemophilia A" OR "Classic Hemophilia" OR haemophilia OR "Autosomal Hemophilia A" OR "Factor VIII Deficiency") OR ("ahf deficiency" OR "ahg deficiency" OR "classic hemophilia" OR "factor viii deficiency" OR "haemophilia a" OR "hemophilia plasma" OR "true haemophilia" OR "true hemophilia") OR ("Hemophilia B" OR "Factor IX Deficiencies" OR "Factor IX Deficiency" OR "Hemophilia B Leyden" OR "Hemophilia B(M) " OR "Plasma Thromboplastin Component Deficiency" OR "F9 Deficiency" OR "Christmas Disease" OR "Haemophilia B" ) OR ("christmas disease" ) OR ("haemophilia (PWH) " OR haemophilia)) AND (("Exercise Therapy" OR "Remedial Exercise" OR "Remedial Exercises" OR "Exercise Therapies" OR "Rehabilitation Exercise" OR "Rehabilitation Exercises") OR ("corrective exercise" OR "exercise movement techniques" OR "exercise treatment" OR "kinesitherapeutic exercises" OR "kinesitherapeutic methodology" OR "kinesitherapeutic treatment" OR kinesitherapy OR "specialized kinesitherapeutic methodology" OR kinesiotherapy) OR ("Physical Therapy Modalities" OR "Physical Therapy Modality" OR "Physiotherapy (Techniques) " OR "Physical Therapy Techniques" OR "Physical Therapy Technique" OR "Group Physiotherapy" OR "Physical Therapy" OR "Neurological Physiotherapy" OR neurophysiotherapy) OR ("physical therapy techniques" OR "physical treatment" OR "physio therapy" OR physiotherapy) OR ("Physical Therapy") OR (exercise OR exercises OR "Physical Activity" OR "Physical Activities" OR "Physical Exercise" OR "Physical Exercises" OR "Acute Exercise" OR "Acute Exercises" OR "Isometric Exercises" OR "Isometric Exercise" OR "Aerobic Exercise" OR "Aerobic Exercises" OR "Exercise Training" OR "Exercise Trainings")) | 780 |
| Scopus | ( ( TITLE-ABS-KEY ( "Hemophilia A" OR "Hemophilia As" OR hemophilia OR "Congenital Hemophilia A" OR "Classic Hemophilia" OR haemophilia OR "Autosomal Hemophilia A" OR "Factor VIII Deficiency" ) OR TITLE-ABS-KEY ( "ahf deficiency" OR "ahg deficiency" OR "classic hemophilia" OR "factor viii deficiency" OR "haemophilia a" OR "hemophilia plasma" OR "true haemophilia" OR "true hemophilia" ) OR TITLE-ABS-KEY ( "Hemophilia B" OR "Factor IX Deficiencies" OR "Factor IX Deficiency" OR "Hemophilia B Leyden" OR "Hemophilia B(M) " OR "Plasma Thromboplastin Component Deficiency" OR "F9 Deficiency" OR "Christmas Disease" OR "Haemophilia B" ) OR TITLE-ABS-KEY ( "christmas disease" ) OR TITLE-ABS-KEY ( "haemophilia (PWH) " OR haemophilia ) ) ) AND ( ( TITLE-ABS-KEY ( "Exercise Therapy" OR "Remedial Exercise" OR "Remedial Exercises" OR "Exercise Therapies" OR "Rehabilitation Exercise" OR "Rehabilitation Exercises" ) OR TITLE-ABS-KEY ( "corrective exercise" OR "exercise movement techniques" OR "exercise treatment" OR "kinesitherapeutic exercises" OR "kinesitherapeutic methodology" OR "kinesitherapeutic treatment" OR kinesitherapy OR "specialized kinesitherapeutic methodology" OR kinesiotherapy ) OR TITLE-ABS-KEY ( "Physical Therapy Modalities" OR "Physical Therapy Modality" OR "Physiotherapy (Techniques) " OR "Physical Therapy Techniques" OR "Physical Therapy Technique" OR "Group Physiotherapy" OR "Physical Therapy" OR "Neurological Physiotherapy" OR neurophysiotherapy ) OR TITLE-ABS-KEY ( "physical therapy techniques" OR "physical treatment" OR "physio therapy" OR physiotherapy ) OR TITLE-ABS-KEY ( "Physical Therapy" ) OR TITLE-ABS-KEY ( exercise OR exercises OR "Physical Activity" OR "Physical Activities" OR "Physical Exercise" OR "Physical Exercises" OR "Acute Exercise" OR "Acute Exercises" OR "Isometric Exercises" OR "Isometric Exercise" OR "Aerobic Exercise" OR "Aerobic Exercises" OR "Exercise Training" OR "Exercise Trainings" ) ) ) | 1.265 |
| Web of Science | "Hemophilia A" OR "Hemophilia As" OR Hemophilia OR "Congenital Hemophilia A" OR "Classic Hemophilia" OR Haemophilia OR "Autosomal Hemophilia A" OR "Factor VIII Deficiency" (Topic) or "ahf deficiency" OR "ahg deficiency" OR "classic hemophilia" OR "factor viii deficiency" OR "haemophilia a" OR "hemophilia plasma" OR "true haemophilia" OR "true hemophilia" (Topic) or "Hemophilia B" OR "Factor IX Deficiencies" OR "Factor IX Deficiency" OR "Hemophilia B Leyden" OR "Hemophilia B(M) " OR "Plasma Thromboplastin Component Deficiency" OR "F9 Deficiency" OR "Christmas Disease" OR "Haemophilia B" (Topic) or "christmas disease" (Topic) or "haemophilia (PWH) " OR haemophilia (Topic) and Preprint Citation Index (Exclude – Database) AND "Exercise Therapy" OR "Remedial Exercise" OR "Remedial Exercises" OR "Exercise Therapies" OR "Rehabilitation Exercise" OR "Rehabilitation Exercises" (Topic) or "corrective exercise" OR "exercise movement techniques" OR "exercise treatment" OR "kinesitherapeutic exercises" OR "kinesitherapeutic methodology" OR "kinesitherapeutic treatment" OR kinesitherapy OR "specialized kinesitherapeutic methodology" OR kinesiotherapy (Topic) or "Physical Therapy Modalities" OR "Physical Therapy Modality" OR "Physiotherapy (Techniques) " OR "Physical Therapy Techniques" OR "Physical Therapy Technique" OR "Group Physiotherapy" OR "Physical Therapy" OR "Neurological Physiotherapy" OR Neurophysiotherapy (Topic) or "physical therapy techniques" OR "physical treatment" OR "physio therapy" OR physiotherapy (Topic) or "Physical Therapy" (Topic) or Exercise OR Exercises OR "Physical Activity" OR "Physical Activities" OR "Physical Exercise" OR "Physical Exercises" OR "Acute Exercise" OR "Acute Exercises" OR "Isometric Exercises" OR "Isometric Exercise" OR "Aerobic Exercise" OR "Aerobic Exercises" OR "Exercise Training" OR "Exercise Trainings" (Topic) and Preprint Citation Index (Exclude – Database) | 1.127 |
| EMBASE | ('hemophilia a'/syn OR 'hemophilia b'/syn OR (haemophilia:ti,ab,kw AND pwh:ti,ab,kw) OR haemophilia:ti,ab,kw) AND ('kinesiotherapy'/syn OR 'physiotherapy'/syn OR 'physical therapy':ti,ab,kw OR 'exercise'/syn) AND ([embase]/lim NOT ([embase]/lim AND [medline]/lim) OR ([medline]/lim NOT ([embase]/lim AND [medline]/lim) NOT ([embase classic]/lim AND [medline]/lim))) | 1.004 |
| Cochrane Library | MeSH descriptor: [Hemophilia A] explode all trees OR ("Hemophilia A" OR "Hemophilia As" OR Hemophilia OR "Congenital Hemophilia A" OR "Classic Hemophilia" OR Haemophilia OR "Autosomal Hemophilia A" OR "Factor VIII Deficiency"):ti,ab,kw OR ("ahf deficiency" OR "ahg deficiency" OR "classic hemophilia" OR "factor viii deficiency" OR "haemophilia a" OR "hemophilia plasma" OR "true haemophilia" OR "true hemophilia"):ti,ab,kw OR MeSH descriptor: [Hemophilia B] explode all trees OR ("Hemophilia B" OR "Factor IX Deficiencies" OR "Factor IX Deficiency" OR "Hemophilia B Leyden" OR "Hemophilia B(M) " OR "Plasma Thromboplastin Component Deficiency" OR "F9 Deficiency" OR "Christmas Disease" OR "Haemophilia B"):ti,ab,kw OR ("christmas disease"):ti,ab,kw OR ("haemophilia (PWH) " OR haemophilia):ti,ab,kw AND MeSH descriptor: [Exercise Therapy] explode all trees OR ("Exercise Therapy" OR "Remedial Exercise" OR "Remedial Exercises" OR "Exercise Therapies" OR "Rehabilitation Exercise" OR "Rehabilitation Exercises"):ti,ab,kw OR ("corrective exercise" OR "exercise movement techniques" OR "exercise treatment" OR "kinesitherapeutic exercises" OR "kinesitherapeutic methodology" OR "kinesitherapeutic treatment" OR kinesitherapy OR "specialized kinesitherapeutic methodology" OR kinesiotherapy):ti,ab,kw OR MeSH descriptor: [Physical Therapy Modalities] explode all trees OR ("Physical Therapy Modalities" OR "Physical Therapy Modality" OR "Physiotherapy (Techniques) " OR "Physical Therapy Techniques" OR "Physical Therapy Technique" OR "Group Physiotherapy" OR "Physical Therapy" OR "Neurological Physiotherapy" OR Neurophysiotherapy):ti,ab,kw OR ("physical therapy techniques" OR "physical treatment" OR "physio therapy" OR physiotherapy):ti,ab,kw OR ("Physical Therapy"):ti,ab,kw OR MeSH descriptor: [Exercise] explode all trees OR (Exercise OR Exercises OR "Physical Activity" OR "Physical Activities" OR "Physical Exercise" OR "Physical Exercises" OR "Acute Exercise" OR "Acute Exercises" OR "Isometric Exercises" OR "Isometric Exercise" OR "Aerobic Exercise" OR "Aerobic Exercises" OR "Exercise Training" OR "Exercise Trainings"):ti,ab,kw | 188 |
| PEDro | "Hemophilia A" AND ("Exercise Therapy" OR "Physical Therapy Modalities" OR "Physical Therapy" OR Exercise)  "Hemophilia B" AND ("Exercise Therapy" OR "Physical Therapy Modalities" OR "Physical Therapy" OR Exercise) | 12  0 |
| CINAHL | TI ( "Hemophilia A" OR "Hemophilia As" OR Hemophilia OR "Congenital Hemophilia A" OR "Classic Hemophilia" OR Haemophilia OR "Autosomal Hemophilia A" OR "Factor VIII Deficiency" ) OR AB ( "Hemophilia A" OR "Hemophilia As" OR Hemophilia OR "Congenital Hemophilia A" OR "Classic Hemophilia" OR Haemophilia OR "Autosomal Hemophilia A" OR "Factor VIII Deficiency" ) OR TI ( "ahf deficiency" OR "ahg deficiency" OR "classic hemophilia" OR "factor viii deficiency" OR "haemophilia a" OR "hemophilia plasma" OR "true haemophilia" OR "true hemophilia" ) OR AB ( "ahf deficiency" OR "ahg deficiency" OR "classic hemophilia" OR "factor viii deficiency" OR "haemophilia a" OR "hemophilia plasma" OR "true haemophilia" OR "true hemophilia" ) OR TI ( "Hemophilia B" OR "Factor IX Deficiencies" OR "Factor IX Deficiency" OR "Hemophilia B Leyden" OR "Hemophilia B(M) " OR "Plasma Thromboplastin Component Deficiency" OR "F9 Deficiency" OR "Christmas Disease" OR "Haemophilia B" ) OR AB ( "Hemophilia B" OR "Factor IX Deficiencies" OR "Factor IX Deficiency" OR "Hemophilia B Leyden" OR "Hemophilia B(M) " OR "Plasma Thromboplastin Component Deficiency" OR "F9 Deficiency" OR "Christmas Disease" OR "Haemophilia B" ) OR TI "christmas disease" OR AB "christmas disease" OR TI ( "haemophilia (PWH) " OR haemophilia ) OR AB ( "haemophilia (PWH) " OR haemophilia ) AND TI ( "Exercise Therapy" OR "Remedial Exercise" OR "Remedial Exercises" OR "Exercise Therapies" OR "Rehabilitation Exercise" OR "Rehabilitation Exercises" ) OR AB ( "Exercise Therapy" OR "Remedial Exercise" OR "Remedial Exercises" OR "Exercise Therapies" OR "Rehabilitation Exercise" OR "Rehabilitation Exercises" ) OR TI ( "corrective exercise" OR "exercise movement techniques" OR "exercise treatment" OR "kinesitherapeutic exercises" OR "kinesitherapeutic methodology" OR "kinesitherapeutic treatment" OR kinesitherapy OR "specialized kinesitherapeutic methodology" OR kinesiotherapy ) OR AB ( "corrective exercise" OR "exercise movement techniques" OR "exercise treatment" OR "kinesitherapeutic exercises" OR "kinesitherapeutic methodology" OR "kinesitherapeutic treatment" OR kinesitherapy OR "specialized kinesitherapeutic methodology" OR kinesiotherapy ) OR TI ( "Physical Therapy Modalities" OR "Physical Therapy Modality" OR "Physiotherapy (Techniques) " OR "Physical Therapy Techniques" OR "Physical Therapy Technique" OR "Group Physiotherapy" OR "Physical Therapy" OR "Neurological Physiotherapy" OR Neurophysiotherapy ) OR AB ( "Physical Therapy Modalities" OR "Physical Therapy Modality" OR "Physiotherapy (Techniques) " OR "Physical Therapy Techniques" OR "Physical Therapy Technique" OR "Group Physiotherapy" OR "Physical Therapy" OR "Neurological Physiotherapy" OR Neurophysiotherapy ) OR TI ( "physical therapy techniques" OR "physical treatment" OR "physio therapy" OR physiotherapy ) OR AB ( "physical therapy techniques" OR "physical treatment" OR "physio therapy" OR physiotherapy ) OR TI "Physical Therapy" OR AB "Physical Therapy" OR TI ( Exercise OR Exercises OR "Physical Activity" OR "Physical Activities" OR "Physical Exercise" OR "Physical Exercises" OR "Acute Exercise" OR "Acute Exercises" OR "Isometric Exercises" OR "Isometric Exercise" OR "Aerobic Exercise" OR "Aerobic Exercises" OR "Exercise Training" OR "Exercise Trainings" ) OR AB ( Exercise OR Exercises OR "Physical Activity" OR "Physical Activities" OR "Physical Exercise" OR "Physical Exercises" OR "Acute Exercise" OR "Acute Exercises" OR "Isometric Exercises" OR "Isometric Exercise" OR "Aerobic Exercise" OR "Aerobic Exercises" OR "Exercise Training" OR "Exercise Trainings" ) | 75 |
| SPORTDiscus | TI ( "Hemophilia A" OR "Hemophilia As" OR Hemophilia OR "Congenital Hemophilia A" OR "Classic Hemophilia" OR Haemophilia OR "Autosomal Hemophilia A" OR "Factor VIII Deficiency" ) OR AB ( "Hemophilia A" OR "Hemophilia As" OR Hemophilia OR "Congenital Hemophilia A" OR "Classic Hemophilia" OR Haemophilia OR "Autosomal Hemophilia A" OR "Factor VIII Deficiency" ) OR TI ( "ahf deficiency" OR "ahg deficiency" OR "classic hemophilia" OR "factor viii deficiency" OR "haemophilia a" OR "hemophilia plasma" OR "true haemophilia" OR "true hemophilia" ) OR AB ( "ahf deficiency" OR "ahg deficiency" OR "classic hemophilia" OR "factor viii deficiency" OR "haemophilia a" OR "hemophilia plasma" OR "true haemophilia" OR "true hemophilia" ) OR TI ( "Hemophilia B" OR "Factor IX Deficiencies" OR "Factor IX Deficiency" OR "Hemophilia B Leyden" OR "Hemophilia B(M) " OR "Plasma Thromboplastin Component Deficiency" OR "F9 Deficiency" OR "Christmas Disease" OR "Haemophilia B" ) OR AB ( "Hemophilia B" OR "Factor IX Deficiencies" OR "Factor IX Deficiency" OR "Hemophilia B Leyden" OR "Hemophilia B(M) " OR "Plasma Thromboplastin Component Deficiency" OR "F9 Deficiency" OR "Christmas Disease" OR "Haemophilia B" ) OR TI "christmas disease" OR AB "christmas disease" OR TI ( "haemophilia (PWH) " OR haemophilia ) OR AB ( "haemophilia (PWH) " OR haemophilia ) AND TI ( "Exercise Therapy" OR "Remedial Exercise" OR "Remedial Exercises" OR "Exercise Therapies" OR "Rehabilitation Exercise" OR "Rehabilitation Exercises" ) OR AB ( "Exercise Therapy" OR "Remedial Exercise" OR "Remedial Exercises" OR "Exercise Therapies" OR "Rehabilitation Exercise" OR "Rehabilitation Exercises" ) OR TI ( "corrective exercise" OR "exercise movement techniques" OR "exercise treatment" OR "kinesitherapeutic exercises" OR "kinesitherapeutic methodology" OR "kinesitherapeutic treatment" OR kinesitherapy OR "specialized kinesitherapeutic methodology" OR kinesiotherapy ) OR AB ( "corrective exercise" OR "exercise movement techniques" OR "exercise treatment" OR "kinesitherapeutic exercises" OR "kinesitherapeutic methodology" OR "kinesitherapeutic treatment" OR kinesitherapy OR "specialized kinesitherapeutic methodology" OR kinesiotherapy ) OR TI ( "Physical Therapy Modalities" OR "Physical Therapy Modality" OR "Physiotherapy (Techniques) " OR "Physical Therapy Techniques" OR "Physical Therapy Technique" OR "Group Physiotherapy" OR "Physical Therapy" OR "Neurological Physiotherapy" OR Neurophysiotherapy ) OR AB ( "Physical Therapy Modalities" OR "Physical Therapy Modality" OR "Physiotherapy (Techniques) " OR "Physical Therapy Techniques" OR "Physical Therapy Technique" OR "Group Physiotherapy" OR "Physical Therapy" OR "Neurological Physiotherapy" OR Neurophysiotherapy ) OR TI ( "physical therapy techniques" OR "physical treatment" OR "physio therapy" OR physiotherapy ) OR AB ( "physical therapy techniques" OR "physical treatment" OR "physio therapy" OR physiotherapy ) OR TI "Physical Therapy" OR AB "Physical Therapy" OR TI ( Exercise OR Exercises OR "Physical Activity" OR "Physical Activities" OR "Physical Exercise" OR "Physical Exercises" OR "Acute Exercise" OR "Acute Exercises" OR "Isometric Exercises" OR "Isometric Exercise" OR "Aerobic Exercise" OR "Aerobic Exercises" OR "Exercise Training" OR "Exercise Trainings" ) OR AB ( Exercise OR Exercises OR "Physical Activity" OR "Physical Activities" OR "Physical Exercise" OR "Physical Exercises" OR "Acute Exercise" OR "Acute Exercises" OR "Isometric Exercises" OR "Isometric Exercise" OR "Aerobic Exercise" OR "Aerobic Exercises" OR "Exercise Training" OR "Exercise Trainings" ) | 45 |
| PROQUEST | (abstract("Hemophilia A" OR "Hemophilia As" OR Hemophilia OR "Congenital Hemophilia A" OR "Classic Hemophilia" OR Haemophilia OR "Autosomal Hemophilia A" OR "Factor VIII Deficiency") OR title("Hemophilia A" OR "Hemophilia As" OR Hemophilia OR "Congenital Hemophilia A" OR "Classic Hemophilia" OR Haemophilia OR "Autosomal Hemophilia A" OR "Factor VIII Deficiency") OR abstract("ahf deficiency" OR "ahg deficiency" OR "classic hemophilia" OR "factor viii deficiency" OR "haemophilia a" OR "hemophilia plasma" OR "true haemophilia" OR "true hemophilia") OR title("ahf deficiency" OR "ahg deficiency" OR "classic hemophilia" OR "factor viii deficiency" OR "haemophilia a" OR "hemophilia plasma" OR "true haemophilia" OR "true hemophilia") OR abstract("Hemophilia B" OR "Factor IX Deficiencies" OR "Factor IX Deficiency" OR "Hemophilia B Leyden" OR "Hemophilia B(M) " OR "Plasma Thromboplastin Component Deficiency" OR "F9 Deficiency" OR "Christmas Disease" OR "Haemophilia B") OR title("Hemophilia B" OR "Factor IX Deficiencies" OR "Factor IX Deficiency" OR "Hemophilia B Leyden" OR "Hemophilia B(M) " OR "Plasma Thromboplastin Component Deficiency" OR "F9 Deficiency" OR "Christmas Disease" OR "Haemophilia B") OR abstract("christmas disease") OR title("christmas disease") OR abstract("haemophilia (PWH) " OR haemophilia) OR title("haemophilia (PWH) " OR haemophilia)) AND ((abstract("Exercise Therapy" OR "Remedial Exercise" OR "Remedial Exercises" OR "Exercise Therapies" OR "Rehabilitation Exercise" OR "Rehabilitation Exercises") OR title("Exercise Therapy" OR "Remedial Exercise" OR "Remedial Exercises" OR "Exercise Therapies" OR "Rehabilitation Exercise" OR "Rehabilitation Exercises") OR abstract("corrective exercise" OR "exercise movement techniques" OR "exercise treatment" OR "kinesitherapeutic exercises" OR "kinesitherapeutic methodology" OR "kinesitherapeutic treatment" OR kinesitherapy OR "specialized kinesitherapeutic methodology" OR kinesiotherapy) OR title("corrective exercise" OR "exercise movement techniques" OR "exercise treatment" OR "kinesitherapeutic exercises" OR "kinesitherapeutic methodology" OR "kinesitherapeutic treatment" OR kinesitherapy OR "specialized kinesitherapeutic methodology" OR kinesiotherapy) OR abstract("Physical Therapy Modalities" OR "Physical Therapy Modality" OR "Physiotherapy (Techniques) " OR "Physical Therapy Techniques" OR "Physical Therapy Technique" OR "Group Physiotherapy" OR "Physical Therapy" OR "Neurological Physiotherapy" OR Neurophysiotherapy) OR title("Physical Therapy Modalities" OR "Physical Therapy Modality" OR "Physiotherapy (Techniques) " OR "Physical Therapy Techniques" OR "Physical Therapy Technique" OR "Group Physiotherapy" OR "Physical Therapy" OR "Neurological Physiotherapy" OR Neurophysiotherapy) OR abstract("physical therapy techniques" OR "physical treatment" OR "physio therapy" OR physiotherapy) OR title("physical therapy techniques" OR "physical treatment" OR "physio therapy" OR physiotherapy) OR abstract("Physical Therapy") OR title("Physical Therapy")) OR (abstract(Exercise OR Exercises OR "Physical Activity" OR "Physical Activities" OR "Physical Exercise" OR "Physical Exercises" OR "Acute Exercise" OR "Acute Exercises" OR "Isometric Exercises" OR "Isometric Exercise" OR "Aerobic Exercise" OR "Aerobic Exercises" OR "Exercise Training" OR "Exercise Trainings") OR title(Exercise OR Exercises OR "Physical Activity" OR "Physical Activities" OR "Physical Exercise" OR "Physical Exercises" OR "Acute Exercise" OR "Acute Exercises" OR "Isometric Exercises" OR "Isometric Exercise" OR "Aerobic Exercise" OR "Aerobic Exercises" OR "Exercise Training" OR "Exercise Trainings"))) | 164 |
| Biblioteca Digital Brasileira de Teses e Dissertações (BDTD) | (Todos os campos:"Hemophilia A" OR "Hemophilia B" E Todos os campos:"Exercise Therapy" OR "Physical Therapy Modalities" OR "Physical Therapy" OR Exercise)  "(Todos os campos:"Hemofilia A" OR "Hemofilia B" E Todos os campos:"Terapia por Exercício" OR "Modalidades de Fisioterapia" OR "Physical Therapy" OR "Exercício Físico")" | 02 |
| Google Acadêmico | ("Hemophilia B" OR "Hemophilia A") AND ("Exercise Therapy" OR "Physical Therapy Modalities" OR "Physical Therapy" OR Exercise) | 06 |
| World Federation of Hemophilia | "Exercise Therapy" OR "Physical Therapy" OR Exercise site:https://wfh.org | 01 |
| National Bleeding Disorders Foundation | "Exercise Therapy" OR "Physical Therapy" OR Exercise site:https://www.bleeding.org | 02 |
| Canadian Hemophilia Society | "Exercise Therapy" OR "Physical Therapy" OR Exercise site:https://www.hemophilia.ca | 02 |
| European Association for Haemophilia and Allied Disorders | "Exercise Therapy" OR "Physical Therapy" OR Exercise site:https://www.eahad.org | 0 |
| Federação Brasileira de Hemofilia | "Terapia por Exercício" OR "Fisioterapia" OR Exercício site:https://hemofiliabrasil.org.br | 0 |

**Appendix 2 - Excluded references**

| Title | Author | Year | Reason |
| --- | --- | --- | --- |
| [Synovectomy in the prevention of recurrent joint bleedings in haemophilia (author's transl)] | Schwägerl et al. | 1976 | Wrong concept |
| A blended physiotherapy intervention for persons with hemophilic arthropathy: Development study | Timmer et al. | 2020 | Wrong concept |
| A case report on a multicentre cooperative rehabilitation programme for inhibitor-positive patients with haemophilia A | Kubota et al. | 2018 | Wrong concept |
| A case study on the effect of physiotherapy on joint health and functional independence in a person with hemophilia who underwent upper limb fasciotomy | Kumar et al. | 2023 | Wrong concept |
| A hemophilic arthropathy patient successfully treated with therapeutic exercise accompanied with a prophylactic infusion of factor VIII a case report | Takakura et al. | 2003 | Wrong concept |
| A modified isokinetic strengthening program for patients with severe hemophilia | Greene & Strickler | 1983 | Wrong concept |
| A total program for the patient with hemophilia. II. Physical therapy aspects related to orthopedic and neurologic residuals of bleeding | Boone | 1966 | Wrong concept |
| Abordagem fisioterápica na dor e na qualidade de vida de um indivíduo com artrite hemofílica. Case report | Jorge et al. | 2016 | Wrong concept |
| Acquired haemophilia A in an elderly patient: A case report of functional recovery through physiotherapy | Tedeschi | 2023 | Wrong concept |
| Adolescents with hemophilic knee arthropathy can improve their gait characteristics, functional ability, and physical activity level through kinect-based virtual reality: A randomized clinical trial | Azab et al. | 2024 | Wrong concept |
| Ageing successfully with haemophilia: A multidisciplinary programme | Boccalandro et al. | 2018 | Wrong concept |
| An advanced stage of ankle arthropathy in a haemophilic patient | Sivrioglu et al. | 2013 | Wrong concept |
| An exercise program adapted for haemophilia patients | Hogea & Mircioaga | 2016 | Wrong concept |
| Analysis of a treatment error in a patient with haemophilia A | Beck & Holzschuh | 2008 | Wrong concept |
| Ankle hemophilic arthropathy: Literature review | Greco et al. | 2021 | Wrong concept |
| Applying World Health Organization 2020 guidelines on physical activity and sedentary behavior to people with hemophilia | de la Corte-Rodriguez et al. | 2021 | Wrong concept |
| Approaches to successful total knee arthroplasty in haemophilia A patients with inhibitors | Konkle et al. | 2002 | Wrong concept |
| Aquatic exercise in patients with haemophilia: Electromyographic and functional results from a prospective cohort study | Feldberg et al. | 2021 | Wrong concept |
| Arthritis in hemophilia | Kisker et al. | 1972 | Wrong concept |
| Articular bleeding in hemophilia | Rodriguez-Merchan et al. | 2016 | Wrong concept |
| Atuação da fisioterapia na força muscular em pacientes com hemofilia | Saldanha Torres et al. | 2022 | Wrong concept |
| Care for children with haemophilia during COVID-19: Data of the PedNet study group | Álvarez-Román & Kurnik | 2021 | Wrong concept |
| Cementless total hip arthroplasty in patient with severe haemophilia A: A case report and literature review | Zhang et al. | 2018 | Wrong concept |
| Chronic arthropathy management in haemophilia: Assessing the impact of a new model of care | d'Young et al. | 2012 | Wrong concept |
| Chronic pain in haemophilia: Assessment and analgesic treatment | de la Corte-Rodriguez & Rodriguez-Merchan | 2023 | Wrong concept |
| Chronic pain in patients with hemophilia: Is it preventable? | Visweshwar et al. | 2020 | Wrong concept |
| Clinical practice guidelines for physiotherapists working with persons with bleeding disorders | Canadian Physiotherapists in Hemophilia Care | 2021 | Wrong concept |
| Comparison of the effect of aquatic exercise therapy and land-based therapeutic exercise on knee muscles' strength and quality of life in patients with knee joint arthropathy due to hemophilia | Mazloum et al. | 2014 | Could not be translated |
| Complications of hemophilia in the elbow: Current management | Rodriguez-Merchan & de la Corte-Rodriguez | 2020 | Wrong concept |
| Comprehensive elements of a physiotherapy exercise programme in haemophilia - a global perspective | Blamey et al. | 2010 | Wrong concept |
| Comprehensive management of chronic pain in haemophilia | Young et al. | 2014 | Wrong concept |
| Conservative management for spontaneous lumbar facet joint hemarthrosis in severe hemophilia A: A rare case report | Lai et al. | 2023 | Wrong concept |
| Conservative physiotherapeutic management of chronic haematomata and haemophilic pseudotumours: Case study and comparison to historical management | D'young | 2009 | Wrong concept |
| Conservative treatment measures in hemophilic arthropathy | Müller et al. | 1999 | Wrong concept |
| Conservative treatment of hemarthrosis for prevention of hemophilic synovitis | Ribbans et al. | 1997 | Wrong concept |
| Controversial subjects in musculoskeletal care of haemophilia: Cross fire | Llinás et al. | 2010 | Wrong concept |
| Current and emerging approaches for pain management in hemophilic arthropathy | Gualtierotti et al. | 2022 | Wrong concept |
| Destination fitness | Canadian Hemophilia Society | 2012 | Wrong concept |
| Does weight reduction in haemophilia lead to a decrease in joint bleeds? | Majumdar et al. | 2012 | Wrong concept |
| Effect of a period of aquatic exercise therapy on the quality of life, anxiety and depression in patients with hemophilia | Kargarfard et al. | 2011 | Could not be translated |
| Effect of designed physiotherapy program on total joint health score in hemophilic children | Hatem et al. | 2017 | Wrong concept |
| Effect of exercise on F VIII-complex: Proportional increase of ristocetin cofactor (Von Willebrand factor) and F VIII-AGN, but disproportional increase of F VIII-AHF | Stibbe | 1977 | Wrong population |
| Effect of partial weight bearing program on functional ability and quadriceps muscle performance in hemophilic knee arthritis | Zaky & Hassan | 2013 | Wrong concept |
| Effect of using treadmill exercise on mobility skills in hemophilic children | Alsakhawi & Alsakhawi | 2017 | Wrong concept |
| Effect of whole body vibration training on quadriceps strength, bone mineral density, and functional capacity in children with hemophilia: A randomized clinical trial | El-Shamy | 2017 | Wrong concept |
| Effective prophylaxis with daily recombinant factor VIIa (rFVIIa-Novoseven) in a child with high titre inhibitors and a target joint | Saxon et al. | 2001 | Wrong concept |
| Effectiveness of a balance training home exercise programme for adults with haemophilia: A pilot study | Hill et al. | 2010 | Wrong concept |
| Effectiveness of an educational physiotherapy and therapeutic exercise program in adult patients with hemophilia: A randomized controlled trial | Cuesta-Barriuso et al. | 2017 | Wrong concept |
| Effectiveness of kinesiologic taping on function and pain in patients with hemophilia A | Kazanci et al. | 2023 | Wrong concept |
| Effectiveness of rehabilitation on pain and function in people affected by hemophilia | Scaturro et al. | 2021 | Wrong concept |
| Effectiveness of two modalities of physiotherapy in the treatment of haemophilic arthropathy of the ankle: A randomized pilot study | Cuesta-Barriuso et al. | 2014 | Wrong concept |
| Effects of a supervised therapeutic exercise program on musculoskeletal health and gait in patients with haemophilia: A pilot study | Deniz et al. | 2022 | Wrong concept |
| Effects of circuit training program on quality of life of children with hemophilia | Malawade et al. | 2020 | Wrong concept |
| Effects of exercise in people with haemophilia: An umbrella review of systematic reviews and meta-analyses | Siqueira et al. | 2019 | Wrong concept |
| Effects of functional therapy program on elbow arthropathy of hemophilia patient: Functional therapy program on elbow arthropathy | Rehman et al. | 2022 | Wrong concept |
| Effects of therapeutic exercise and hydrotherapy on pain severity and knee range of motion in patients with hemophilia: A randomized controlled trial | Mazloum et al. | 2014 | Wrong concept |
| Efficacy of pulsed electromagnetic field on hemarthrotic knee in haemophilic adolescence | Heneidy et al. | 2021 | Wrong concept |
| Efficacy of pulsed high-intensity laser therapy on pain, functional capacity, and gait in children with haemophilic arthropathy | El-Shamy & Abdelaal | 2018 | Wrong concept |
| Emicizumab como tratamiento preventivo del sangrado en un paciente con hemofilia A severa X1 | Forteza-Cáceres et al. | 2022 | Wrong concept |
| Establishing an online physical exercise program for people with hemophilia | Wagner et al. | 2019 | Wrong concept |
| Exercício físico e hemofilia: Conceitos e intervenção | Andery et al. | 2012 | Wrong concept |
| Exercise and hemophilia | Wilmott | 2008 | Wrong concept |
| Exercise may decrease further destruction in the adult haemophilic joint | Harris & Boggio | 2006 | Wrong concept |
| Exercise variables and pain threshold reporting for strength training protocols in people with haemophilia: A systematic review of clinical trials | Cruz-Montecinos et al. | 2023 | Wrong concept |
| Exercises for people with hemophilia | Mulder | 2006 | Wrong concept |
| Favourable impact of regular swimming in young people with haemophilia: Experience derived from 'Desafio del Caribe' project | Boadas et al. | 2015 | Wrong concept |
| First case report on persistent patellar dislocation in haemophilia A patient with a factor VIII inhibitor | Chandoga et al. | 2022 | Wrong concept |
| Fitness enhances psychosocial well-being and self-confidence in young men with hemophilia: Results from Project GYM | Khair et al. | 2021 | Wrong concept |
| Five in one therapy for graded treatment of haemophilic arthritis | Liu et al. | 2016 | Wrong concept |
| Flexion contractures of the knee in haemophilia | Silva & Luck | 2007 | Wrong concept |
| From top to toe: An alternative approach to physiotherapy for hemophiliacs | Desmarres & Laurian | 1984 | Wrong concept |
| Functional recovery after bleeding episodes in haemophilia | de Kleijn et al. | 2004 | Wrong concept |
| Gesundheitsförderung für kinder und jugendliche mit hämophilie - gesundheitsberatung, adjuvante bewegungs - therapie und schulsport | Sondermann et al. | 2017 | Wrong concept |
| Guideline for the treatment of haemophilia in South Africa | Mahlangu & Gilham | 2008 | Wrong concept |
| Guidelines for the management of acute joint bleeds and chronic synovitis in haemophilia: A United Kingdom Haemophilia Centre Doctors' Organisation (UKHCDO) guideline | Hanley et al. | 2017 | Wrong concept |
| Guidelines for the management of hemophilia | Srivastava et al. | 2013 | Wrong concept |
| Haemophilia | Getaz et al. | 1977 | Wrong concept |
| Haemophilia & Exercise Project (HEP): The impact of 1-year sports therapy programme on physical performance in adult haemophilia patients | Czepa et al. | 2013 | Wrong concept |
| Haemophilia A and B as a cause for secondary osteoporosis and increased fracture risk | Anagnostis et al. | 2015 | Wrong concept |
| Haemophilia and fragility fractures: From pathogenesis to multidisciplinary approach | Alito et al. | 2023 | Wrong concept |
| Haemophilia and joint disease: Pathophysiology, evaluation, and management. | Knobe & Berntorp | 2011 | Wrong concept |
| Haemophiliac knee: Role of physiotherapy | Khriesat et al. | 2000 | Wrong concept |
| Haemophilic ankle arthropathy: Case reports and review of the literature | Pelzer et al. | 2020 | Wrong concept |
| Haemophilic arthropathy | Mahajan et al. | 2005 | Wrong concept |
| Haemophilic arthropathy in haemophilia patients with inhibitors: New perspectives | Rodriguez-Merchan | 2003 | Wrong concept |
| Haemophilic arthropathy of the elbow | Utukuri & Goddard | 2005 | Wrong concept |
| Haemophilic arthropathy: Can it be prevented? | Parker et al. | 2016 | Wrong concept |
| Haemophilic arthropathy: Contemporary management challenges and a future scenario | Rodriguez-Merchan | 2021 | Wrong concept |
| Hemokinect: A microsoft kinect V2 based exergaming software to supervise physical exercise of patients with hemophilia | Mateo et al. | 2018 | Wrong concept |
| Hemophilia and Exercise Project (HEP) conception and contents of a programmed sports therapy for hemophilic patients | Scharrer et al. | 2008 | Wrong concept |
| Hemophilia and resistance training: Implications for the strength and conditioning professional | Coelho & Cameron | 1999 | Wrong concept |
| Hemophilic hemarthroses: Diagnosis and management | Rodríguez-Merchán & de la Corte-Rodríguez | 2015 | Wrong concept |
| Hemophilic pseudotumor of the distal parts of the radius and ulna: A case report | Shaheen & Alasha | 2005 | Wrong concept |
| Heterotopic ossification in quadratus femoris muscle in a haemophilic patient | Kalenderer et al. | 2012 | Wrong concept |
| High-impact physical activity increases bleeding risk in children with hemophilia | Stapleton | 2012 | Wrong concept |
| Homo erectus haemophilus - the haemophilic arthropathy of the leg | Taubner et al. | 2011 | Wrong concept |
| How we treat: Considerations for physiotherapy in the patient with haemophilia and inhibitors undergoing elective orthopaedic surgery. | Forsyth & Zourikian | 2012 | Wrong concept |
| Iatrogenic fracture of the proximal tibia as a complication of knee manipulation under anaesthesia in a haemophilia patient with an ipsilateral stiff knee secondary to a supracondylar non-union of the femur | Rodriguez-Merchan et al. | 2012 | Wrong concept |
| Iilizarov external wxator for bilateral severe xexion deformity of the knee in haemophilia: Case report | Kumar et al. | 2010 | Wrong concept |
| Iliopsoas haemorrhage in patients with haemophilia: Results from one centre | Balkan et al. | 2005 | Wrong concept |
| Impact of exercise on hemophilia | Zetterberg et al. | 2018 | Wrong concept |
| Implementation and assessment of a self- and community-based rehabilitation programme in patients with haemophilia from Côte d’Ivoire | Lobet et al. | 2019 | Wrong concept |
| Improving care of older patients with hemophilia during COVID-19 pandemic, reducing the risk of venous thrombosis with home exercises | Demirci et al. | 2022 | Wrong concept |
| In-hospital rehabilitation after multiple joint procedures of the lower extremities in haemophilia patients: Clinical guidelines for physical therapists | de Kleijn et al. | 2011 | Wrong concept |
| In-patient rehabilitation in haemophilic subjects with total knee arthroplasty | Viliani et al. | 2011 | Wrong concept |
| Individual and combined efficacy of multi angel isometric exercises and electrical stimulation in treatment of haemophilic arthritis | ElKhozamy et al. | 2019 | Wrong concept |
| Influência do tratamento fisioterapêutico na independência funcional de pessoas com hemofilia submetidas à sinovectomia radioativa | Silva et al. | 2023 | Wrong concept |
| Intelligent game engines for home exercises (exergames) in boys with haemophilia | Boccalandro et al. | 2021 | Wrong concept |
| Joint contractures in the hemophilias | Atkins et al. | 1987 | Wrong concept |
| Knee arthropathy: When things go wrong | Solimeno et al. | 2012 | Wrong concept |
| Maintenance of musculoskeletal function in people with haemophilia | Battistella | 1998 | Wrong concept |
| Management of arthrofibrosis in haemophilic arthropathy | Solimeno et al. | 2010 | Wrong concept |
| Management of fixed flexion contracture of the elbow in haemophilia | Gilbert & Radomisli | 1999 | Wrong concept |
| Management of haemophilia and its complications in developing countries | Ghosh | 2004 | Wrong concept |
| Management of haemophilic arthropathy | Bossard et al. | 2008 | Wrong concept |
| Management of muscle haematomas in patients with severe haemophilia in an evidence-poor world | Sørensen et al. | 2012 | Wrong concept |
| Management of musculoskeletal problems of hemophilia | Boone | 1974 | Wrong concept |
| Management of the elbow joint | Heim et al. | 2012 | Wrong concept |
| Manual and educational therapy in the treatment of hemophilic arthropathy of the elbow: A randomized pilot study | Cuesta-Barriuso et al. | 2018 | Wrong concept |
| MASAC recommendations regarding physical therapy management for the care of persons with bleeding disorders | National Hemophilia Foundation | 2023 | Wrong concept |
| Modern management of haemophilic arthropathy. | Raffini & Manno | 2007 | Wrong concept |
| Multidiciplinary treatment of a haemophilic person with total hip replacement surgery - a case report | Nikolikj-Dimitrova et al. | 2013 | Wrong concept |
| Muscle rehabilitation in haemophilia | Beeton et al. | 1998 | Wrong concept |
| Muscle strengthening intervention for boys with haemophilia: Developing and evaluating a best-practice exercise programme with boys, families and health-care professionals | Hashem et al. | 2020 | Wrong concept |
| Muscular complications of hemophilia | Alcalay | 2009 | Wrong concept |
| Musculoskeletal aspects of haemophilia | Rodriguez-Merchan et al. | 2008 | Wrong concept |
| Musculoskeletal complications of haemophilia: The joint | N/A | 2000 | Wrong concept |
| Musculoskeletal pain and functional ability in haemophilia A and B. Physiotherapy and rehabilitation in haemophilia patients | Santavirta et al. | 2001 | Wrong concept |
| Musculoskeletal problems in persons with inhibitors: How do we treat? | Caviglia et al. | 2012 | Wrong concept |
| Musculoskeletal treatment in haemophilia | Atilla & Güney-Deniz | 2019 | Wrong concept |
| New concepts regarding articular cartilage preservation in persons with haemophilia | Heim & Wallny | 2007 | Wrong concept |
| Non-haematological pathologies - 'the importance of musculoskeletal assessment in haemophilia' | Dalzell | 2005 | Wrong concept |
| Non-surgical correction of knee flexion deformity in persons with haemophilia: A staged multidisciplinary approach | Tilak et al. | 2024 | Wrong concept |
| Obesity and physical exercise in hemophilia | Nieva | 2023 | Wrong concept |
| Operative treatment of anterior heterotopic bone formation of the elbow in a patient with severe haemophilia A | Mortazavi et al. | 2006 | Wrong concept |
| Optimal management of hemophilic arthropathy and hematomas. | Lobet et al. | 2014 | Wrong concept |
| Orthopaedic surgery in haemophilia patients with inhibitors: A practical guide to haemostatic, surgical and rehabilitative care | Teitel et al. | 2009 | Wrong concept |
| Osteoporosis management and falls prevention in patients with haemophilia: Review of haemophilia guidelines | Petkovic et al. | 2022 | Wrong concept |
| Pain and pain management in haemophilia | Auerswald et al. | 2016 | Wrong concept |
| Pathogenesis and treatment of osteoporosis in patients with hemophilia | Lin et al. | 2023 | Wrong concept |
| Pedobarographic and stabilometric analysis and exercises for children with recurrent ankle haemarthrosis | Elnaggar | 2018 | Wrong concept |
| Perioperative physiotherapy for total ankle replacement in patients with inherited bleeding disorders: Outline of an algorithm | Kotela et al. | 2017 | Wrong concept |
| Peripheral nerve injuries in haemophilia | Rodriguez-Merchan | 2014 | Wrong concept |
| Physical activity recommendations for children with specific chronic health conditions: Juvenile idiopathic arthritis, hemophilia, asthma and cystic fibrosis | Philpott et al. | 2010 | Wrong concept |
| Physical rehabilitation in haemophilic arthropathy: systematic review and pain-related meta-analysis | Pacheco-Serrano et al. | 2021 | Wrong concept |
| Physical therapy and hemophilia | Raymond & Fondanesche | 2009 | Wrong concept |
| Physical therapy for a patient with christmas disease. | Rutan | 1963 | Wrong concept |
| Physical therapy for end-stage hemophilic arthropathy: a case report | Gao et al. | 2023 | Wrong concept |
| Physical training increases isometric muscular strength and proprioceptive performance in haemophilic subjects | Hilberg et al. | 2003 | Wrong concept |
| Physiotherapy after Total Ankle Replacement in a patient with haemophilia - a case study | Gleb et al. | 2015 | Wrong concept |
| Physiotherapy evaluation and intervention in the acute hemarthrosis: Challenging the paradigm | Zourikian & Forsyth | 2011 | Wrong concept |
| Physiotherapy following elective orthopaedic procedures | de Kleijn et al. | 2006 | Wrong concept |
| Physiotherapy for adult patients with haemophilia | Beeton | 2008 | Wrong concept |
| Physiotherapy for prevention and treatment of chronic hemophilic synovitis | Buzzard | 1997 | Wrong concept |
| Physiotherapy for the prevention of articular contraction in haemophilia | Buzzard | 1999 | Wrong concept |
| Physiotherapy for the treatment of articular contractures in haemophilia | Heijnen & de Kleijn | 1999 | Wrong concept |
| Physiotherapy home exercise program for haemophiliacs | Pierstorff et al. | 2011 | Wrong concept |
| Physiotherapy in patients with congenital haemorrhagic diathesis in the material of the systemic rehabilitation department | Bukowska et al. | 2021 | Wrong concept |
| Physiotherapy in the management of hemophilia | Beeton & Padkin | 2010 | Wrong concept |
| Physiotherapy interventions for pain management in haemophilia: A systematic review | McLaughlin et al. | 2020 | Wrong concept |
| Physiotherapy management of haemophilia | Buzzard & Beeton | 2001 | Wrong concept |
| Physiotherapy management of haemophilia in children | Buzzard | 2008 | Wrong concept |
| Physiotherapy of children with hemophilia | Fiodorenko-Dumas et al. | 2009 | Wrong concept |
| Physiotherapy treatment in patients with hemophilia and chronic ankle arthropathy: A systematic review | Cuesta-Barriuso et al. | 2013 | Wrong concept |
| Pohybová aktivita pacientů s hemofilií. | Pokorný & Slabý | 2016 | Wrong concept |
| Prática de esportes e exercícios: orientações básicas para hemofílicos | Silveira et al. | 2014 | Wrong concept |
| Prevention and treatment of joint problems in children with haemophilia | Heijnen & Helders | 2007 | Wrong concept |
| Prevention of haemophilic arthropathy during childhood. May common orthopaedic management be extrapolated from patients without inhibitors to patients with inhibitors? | Rodriguez-Merchan et al. | 2008 | Wrong concept |
| Programmed Sports Therapy (PST) in People with Haemophilia (PwH) "sports Therapy Model for Rare Diseases" | Hilberg | 2018 | Wrong concept |
| Project GYM: A randomized feasibility study investigating effect on motivation of personal trainer-led exercise in young men with hemophilia | McLaughlin et al. | 2021 | Wrong concept |
| Promoting pain coping skills in haemophilia: A remote intervention integrating exercise and pain education | Feldberg et al. | 2024 | Wrong concept |
| Proof of concept and feasibility of a blended physiotherapy intervention for persons with haemophilic arthropathy | Timmer et al. | 2022 | Wrong concept |
| Proprioceptive training in haemophilia. | Buzzard | 1998 | Wrong concept |
| Protocol for a feasibility randomised controlled trial of a musculoskeletal exercise intervention versus usual care for children with haemophilia | Hashem et al. | 2019 | Wrong concept |
| Pulsed Nd:YAG laser: effects on pain, postural stability, and weight-bearing pattern in children with hemophilic ankle arthropathy | Elnaggar | 2020 | Wrong concept |
| Reabilitação na artropatia hemofílica | Gomes et al. | 2018 | Wrong concept |
| Recent advances in musculoskeletal physiotherapy for haemophilia | Stephensen, D.; Bladen, M.; McLaughlin, P. | 2018 | Wrong concept |
| Rehabilitation and nonsurgical management of musculoskeletal problems in the hemophilic patient | Dietrich | 1975 | Wrong concept |
| Rehabilitation and physiotherapy for adults with haemophilia | Querol & Beeton | 2007 | Wrong concept |
| Rehabilitation following ankle surgery in haemophilia | Buzzard & Briggs | 2007 | Wrong concept |
| Rehabilitation improved walking ability for three haemophilia patients with inhibitors | Shimokawa & Takedani | 2014 | Wrong concept |
| Rehabilitation in haemophilia - Options in the developing world | Battistella | 1998 | Wrong concept |
| Rehabilitation in haemophilic children with inhibitors using recombinant activated factor VII | Janić et al. | 2008 | Wrong concept |
| Rehabilitation management of a triceps surae muscle injury in a young male with haemophilia A and high-titre inhibitors | Frizziero et al. | 2021 | Wrong concept |
| Rehabilitation of joint surgery in hemophiliacs | de la Corte-Rodríguez et al. | 2015 | Wrong concept |
| Rehabilitation of patients with haemophilia after orthopaedic surgery: A case study | Stephensen | 2005 | Wrong concept |
| Results of feasibility and safety of randomised controlled trial of a musculoskeletal exercise intervention versus usual care for children with haemophilia | Bladen et al. | 2020 | Wrong concept |
| Role of exercise and physical activity on haemophilic arthropathy, fall prevention and osteoporosis | Forsyth et al. | 2011 | Wrong concept |
| Role of physiotherapy in haemophilia - an evidence based practice | Panchasara et al. | 2016 | Wrong concept |
| Safety and effectiveness of telerehabilitation program in people with severe haemophilia in Chile. A qualitative study | Aliaga-Castillo et al. | 2022 | Wrong concept |
| Secondary prophylaxis vs. on-demand treatment to improve quality of life in severe adult haemophilia A patients: A prospective study in a single centre | Aznar et al. | 2014 | Wrong concept |
| Self-monitoring has potential for home exercise programmes in patients with haemophilia | Goto et al. | 2014 | Wrong concept |
| Sequential treatment with bolus and continuous infusion of recombination factor VIIa for hip arthroplasty in a patient with haemophilia A and inhibitor | Perez et al. | 2002 | Wrong concept |
| Short-term evaluation of synovectomy in haemophilia | Pietrogrande & Mannucci | 1972 | Wrong concept |
| Shoulder hemiarthroplasty to manage haemophilic arthropathy: Two case studies | Dalzell | 2004 | Wrong concept |
| Sports and exercise in haemophilia: Benefits and challenges | Riske | 2007 | Wrong concept |
| Stop only advising physical activity in adults with haemophilia… prescribe it now! The role of exercise therapy and nutrition in chronic musculoskeletal diseases | Lobet et al. | 2016 | Wrong concept |
| Strategies to encourage physical activity in patients with hemophilia to improve quality of life | Goto et al. | 2016 | Wrong concept |
| Successful concurrent triple surgery in an adolescent patient with haemophilia A and inhibitors treated with recombinant factor VIIa [2] | Janic et al. | 2007 | Wrong concept |
| Successful total hip replacement in a patient with severe haemophilia A with inhibitors using recombinant factor VIIa | Pasa et al. | 2008 | Wrong concept |
| Synovectomy and continuous passive motion (CPM) in hemophiliac patients | Limbird & Dennis | 1987 | Wrong concept |
| Tai Chi Chuan for people with haemophilia [1] | Danusantoso & Heijnen | 2001 | Wrong concept |
| The acute effect of moderate intensity aquatic exercise on coagulation factors in haemophiliacs | Beltrame et al. | 2015 | Wrong concept |
| The benefits of exercise for patients with haemophilia and recommendations for safe and effective physical activity | Negrier et al. | 2013 | Wrong concept |
| The effect of 8-weeks exercise in water on factor VIII and partial thromboplastin time (PTT) of men with hemophilia | Soltani et al. | 2016 | Could not be translated |
| The effect of a home exercise program for patients with hemophilia | Goto et al. | 2014 | Wrong concept |
| The effect of an exercise intervention on aerobic fitness, strength and quality of life in children with haemophilia (ACTRN012605000224628) | Broderick et al. | 2006 | Wrong concept |
| The effect of Nordic Walking on joint status, quality of life, physical ability, exercise capacity and pain in adult persons with haemophilia | Salim et al. | 2016 | Wrong concept |
| The effect of tai chi self help group program for hemophilic arthritis patients z1 혈우병관절염 수술환자를 위한 타이치자조관리 프로그램 효과 | Kang et al. | 2012 | Wrong concept |
| The effects of an exercise therapy program on joint range of motion, aerobic fitness, and anxiety of hemophilia A patients | Firoozabadi et al. | 2012 | Could not be translated |
| The effects of exercise training on physical activity level, daily living activities, and participation in children with hemophilia | Atay et al. | 2023 | Wrong concept |
| The effects of manual therapy and exercises on pain, muscle strength, joint health, functionality and quality of life in haemophilic arthropathy of the elbow joint: A randomized controlled pilot study | Tat et al. | 2021 | Wrong concept |
| The Ilizarov technique in the management of haemophilic knee flexion contracture | Wei et al. | 2023 | Wrong concept |
| The impact of a specific aqua-training for adult haemophilic patients - results of the WATERCISE study (WAT-QoL) | von Mackensen et al. | 2012 | Wrong concept |
| The management of acute haemophilic haemarthroses and muscle haematoma | Lurie & Bailey | 1972 | Wrong concept |
| The management of knee flexion contractures in haemophilia: Brief report. | Nelson et al. | 1989 | Wrong concept |
| The role of physical medicine and rehabilitation in haemophiliac patients | de la Corte-Rodriguez & Rodriguez-Merchan | 2013 | Wrong concept |
| The role of physiotherapy after total knee arthroplasty in patients with haemophilia | Lobet et al. | 2008 | Wrong concept |
| The use of plasma from exercised donors in haemophilia: Preliminary report | Taylor & Sise | 1967 | Wrong concept |
| The Utrecht approach to exercise in chronic childhood conditions: The decade in review | van Brussel et al. | 2011 | Wrong concept |
| Theraband® exercises with music for persons with haemophilia | Babu et al. | 2013 | Wrong concept |
| Therapeutic choices in persons with haemophilia at the time of COVID-19 | Coppola et al. | 2020 | Wrong concept |
| Therapeutic options for hemarthrosis in severe hemophilic patients | Costa et al. | 2022 | Wrong concept |
| Therapeutic options in the management of hemophilic synovitis | Gilbert & Radomisli | 1997 | Wrong concept |
| Therapeutic value of kinesio taping in reducing lower back pain and improving back muscle endurance in adolescents with hemophilia | Azab et al. | 2020 | Wrong concept |
| Total knee arthroplasty in hemophiliacs: Gains in range of motion realized beyond twelve months postoperatively | Kamath et al. | 2012 | Wrong concept |
| Total knee arthroplasty in patients with hemophilia: What do we know? | Mortazavi et al. | 2020 | Wrong concept |
| Total knee replacement with and without emicizumab: A unique comparison of perioperative management | Evans et al. | 2020 | Wrong concept |
| Trapezoid supracondylar femoral extension osteotomy for knee flexion contractures in patients with haemophilia | Mortazavi et al. | 2008 | Wrong concept |
| Traumatic hemarthrosis of the knee secondary to hemophilia A in a collegiate soccer player: A case report | Fiala et al. | 2002 | Wrong concept |
| Treatment of Hæmophilia | - | 1973 | Wrong concept |
| Treatment of haemophilia: Building on strength in the third millennium | Mannucci | 2011 | Wrong concept |
| Two haemophilia patients with inhibitors who became ambulatory after physiotherapy under haemostatic cover with bypassing agents. | Kajiwara et al. | 2013 | Wrong concept |
| Upper-body exercises with external resistance are well tolerated and enhance muscle activity in people with hemophilia | Calatayud et al. | 2019 | Wrong concept |
| Using evidence-based co-design to develop a hybrid delivered exercise intervention that aims to increase confidence to exercise in people with haemophilia | Taylor et al. | 2023 | Wrong concept |
| Using theory of change to co-create a programme theory for a telerehabilitation intervention for pain management in people with haemophilia | McLaughlin et al. | 2023 | Wrong concept |

**Appendix 3 - Overview of interventions, exercise programmes and results.**

| Author (year)  Design | Intervention | Exercise programmes | Results | Bleeding during the intervention |
| --- | --- | --- | --- | --- |
| Pelletier et al. (1987)  Case report | Supervised isometric exercise programme (3 w, 9* sessions) *8 sessions were completed | Isometric exercise: quadriceps and hamstrings at 45°, 60° and 90° of knee flexion (10 reps/10 s contraction/10 s rest between reps/two thirds MVIC) | Significant improvement in muscle strength (95% CI) for all measurements except 60° after the intervention, without significantly affecting ROM, knee circumference and skin temperature, and without causing bleeding or discomfort. | No episodes |
| Tiktinsky et al. (2002)  Prospective and retrospective pilot | Supervised exercise programme (3 d/w, 45 to 60 minutes). Participant 1: 24 m. Participant 2: 12 m | Stretching: calf, hamstrings, hip adductors, lower back, neck rotators and upper limbs (5 to 15 min)  Strengthening exercise: free weights - individualised program (30 to 45 min) (intensity based on maximum reps without load - progression by up to 10% per session up to 150 to 250 reps without load). Participant 1: shoulder (flexion, abduction, external and internal rotation), elbow (flexion-extension) and forearm (pronation-supination) (20 to 40 reps). Participant 2: hip (flexion-extension and abduction-adduction), knee (flexion-extension) and ankle (dorsiflexion-plantar flexion) (20 to 220 reps) | Improvements in muscle strength, especially in the muscle groups of the respective target joints (elbow and knee), bleeding frequency and associated pain. | No increase in bleeding |
| Gomis et al. (2009)  Systematic review | Medline, CINAHL, Embase and SportDiscus (up to April 2007): 103 studies (29 experimental, 27 observational, 47 clinical trials) were included. IG: exercise or sports programmes. CG: no intervention (n=3), no haemophilia (n=1) (n=experimental studies) | Sport (n=5)  Strengthening exercise (n=14)  Mobility/ROM exercise (n=1)  Proprioceptive exercise (n=3)  Aerobic exercise (n=2)  Aquatic exercise (n=3)  Postural and coordination training (n=1)  Unspecified modality (n=5) (n=experimental studies) | The included studies had methodological flaws. Even experimental studies with better methodological quality presented limitations in relation to their sample size, materials used and description of exercise programmes. Overall, regular physical exercise showed positive effects on PwH, including improving muscle strength and coordination, preventing muscle contractures, and reducing pain. | N/A |
| Vallejo et al. (2010)  Single-arm prospective | Supervised aquatic exercise programme (9 w, 3 d/w, 60 min/d) | Endurance training: free active or floaters - shoulders (flexion-extension and abduction-adduction) and elbows (flexion-extension) (2 sets/20 reps/30 s active rest/intensity 2 to 7 on the modified Robertson scale - progressive increase in intensity)  Strengthening exercise: free active or floaters - horizontal and vertical traction movements (2 sets/30 s/2 min active rest/intensity 2 to 7 on the modified Robertson scale - progressive increase in intensity)  Aerobic exercise: 4 swimming exercises (20 min) (50 meters each/50 to 75% MHR - progressive increase in intensity)  Stretching: upper limbs and lower limbs (2 sets/30 s) | Significant improvement (p≤.008) in oxygen consumption, relative oxygen consumption, carbon dioxide, respiratory quotient and distance covered in the Cooper test after the intervention. | No episodes |
| Mulvany et al. (2010)  Single arm pre-test-post-test | Individualised supervised exercise programme (6 w, 2 d/w) | Strengthening exercise: major muscle groups (isotonic or isometric, depending on pain or joint impairment) and functional strengthening activities (walking, sitting and standing, and climbing stairs) (1 to 3 sets/10 to 20 reps/5 to 10 s concentric contraction/40 to 75% Nicholas dynamometry isometric muscle test - progression by 5 to 10% per week if no adverse effects reported)  Stretching: gentle, low load and prolonged (2 to 20 min)  Aerobic exercise: low impact, depending on the participant's preference (50 to 70% MHR - progression by 5 to 10% if no adverse effects are reported) | Significant improvement in ROM (p≤.05), muscle strength (p≤.01), upper limb circumference (p≤.05) and distance covered in 6 minutes (p<.01) after the intervention. | No episodes |
| Querol et al. (2011)  Systematic review | Web of Science, PubMed, SportDiscus and Scopus (up to 2010): 74 studies were included. IG: exercise or sports programmes. CG: no haemophilia (n=2), no arthropathy (n=1), unspecified (n=3) (n=experimental studies) | Sport (n=2)  Strengthening exercise (n=12)  Strengthening + Strengthening exercise (n=2)  Flexibility exercise (n=1)  Proprioception exercise (n=1)  Aerobic exercise (n=4)  Aquatic exercise (n=2)  Postural training (n=1)  Balance training (n=1)  Gait training (n=1)  Unspecified modality (n=1) (n=experimental studies) | Overall, regular physical exercise and sports have positive effects on PwH, bringing physical and psychosocial benefits, including improved muscle strength, aerobic capacity, self-esteem, socialisation and, consequently, quality of life. There is a consensus on the need to include physiotherapy, exercise and sport programmes in the management of PwH, considering the physical, emotional and social benefits. Physical exercise and sport are also recommended for people with inhibitors. | N/A |
| Souza et al. (2012)  Systematic review | Medline and Highwire (up to 2012, ≤ 30 y since publication): 8 studies were included. IG: exercise programmes (3 w to 2 y, 2 to 7 d/w). CG: unspecified (n=8) | Aerobic exercise (n=4) (intensity defined by FCM [n=3], N/A [n=1])  Strengthening training (n=6) (intensity defined by MR [n=1], MIF [n=2]), N/A [n=3]) | Most exercise interventions have shown positive effects in PwH. Aerobic exercise combined with resistance training seem to improve functional capacity and ROM, although the intensity required to increase clotting factors has not yet been determined. Resistance training, whether isolated or combined with aerobic exercise, seem to increase muscle mass and strength, ROM and proprioception of the lower limbs, improving functional capacity and reducing bleeding episodes. A large number of exercises or heavy loads are not necessary. In conclusion, exercise in PwH brings benefits to physical fitness and coagulation mechanisms, suggesting its application as an adjuvant therapy to conventional treatment. | N/A |
| Kargarfard et al. (2013)  Non-RCT | IG: supervised aquatic exercise programme (8 w, 3 d/w, 40 to 60 min/d). CG: no intervention | Aquatic exercise: warm-up (progressive aerobic activity, unspecified) (50 to 74% MHR), 10 simple movements - 5 for upper limbs and 5 for lower limbs (1 min per movement/maximum intensity or until tolerable in the presence of pain) and cool-down (flexibility exercise, unspecified) (5 min) | Compared to the CG, IG showed a significant improvement (p<.05) in muscle strength and ROM in all joints after the intervention. | N/A |
| Eid et al. (2014)  RCT | Both: supervised exercise programme (12 w, 3 d/w, 60 min/d). IG: endurance training (12 w, 3 d/w, 40 min/d) | Stretching: biceps brachii, hamstrings and calves (5 reps/20 s stretching/20 s relaxation)  Isometric exercise: quadriceps, hamstrings, tibialis anterior, calves, biceps and triceps (5 to 10 reps/5 s contraction/5 s relaxation)  Aerobic exercise: treadmill - warm-up (5 min), walking at 75% of comfortable speed and 0° incline (20 min) and cool-down (5 min)  Endurance training: bicycle ergometer - warm-up (pedalling at a lower intensity) (5 min), pedalling with a gradual increase in load (10 min) and cool-down (pedalling without load) (5 min); free weights - quadriceps and hamstrings (3 sets/10 to 15 reps/2 min rest/2 to 6 kg - progression applied individually) | Significant improvement in functional capacity in both groups after the intervention (p=.0001). IG showed significant improvement in bone mineral density (p=.001) and muscle strength (p=.0001) after the intervention. IG showed better results compared to CG (p<.05). | N/A |
| Parhampour et al. (2014)  RCT | Supervised exercise programme (6 w, 3 d/w, 30 to 40 min/d). RT: strengthening exercise + placebo pulsed electromagnetic fields. RTPEMF: strengthening exercise + pulsed electromagnetic fields. PEMF: pulsed electromagnetic fields only. CG: no intervention | Strengthening exercise: free weights, stationary resistance equipment - hips (flexion, abduction and extension), knees (extension), chest and shoulder press, scapula retraction, leg press, spine extension and squats (RT: 10 to 15 reps. RTPEMF: 5 to 10 reps/10 s rest between reps/1 to 2 min rest between exercises/50 to 60% 1RM - progression every 2 w) | Compared to the CG, the RT and RTPEMF groups showed significant improvement in bone alkaline phosphatase (p≤.05) after the intervention. Compared to the CG and the PEMF group, the absolute changes in the modified Colorado Questionnaire total score were significant for the knee, ankle and elbow joints in the RT and RTPEMF groups after the intervention (p≤.05). | N/A |
| Al-Sharif et al. (2014)  RCT | Supervised aerobic training programme (12 w, 3 d/w, 30 min/d). A: moderate intensity. B: mild intensity | Aerobic exercise: treadmill (Bruce Protocol) - warm-up (5 min), training (30 min) (A: 65 to 75% MHR. B: 50 to 60% MHR) and cool-down (5 min) | Significant improvement (p<.001) in bone metabolism markers and handgrip strength in both groups after the intervention. Training at moderate intensity showed better results compared to training at light intensity (p<.001). | N/A |
| Mohamed & Sherief (2015)  RCT | Both: supervised exercise programme (12 w, 3 d/w, 60 min/d). A: bicycle ergometer. B: treadmill | Stretching: biceps brachii, hamstrings and calves (5 reps/20 s stretching/20 s relaxation)  Isometric exercise: quadriceps, hamstrings, tibialis anterior, calves, biceps brachii and triceps brachii (5 reps/5 s contraction/5 s relaxation)  Balance training (unspecified) and gait training with obstacles  Aerobic exercise: warm-up (5 min), training (20 min) (A: cycling with gradual increase in load. B: walking at 75% speed relative to the ground and 0° inclination) and cool-down (5 min) (A: pedalling without load. B: stretching and walking) | Significant improvement (p<.05) in balance and gait in both groups after the intervention. Treadmill training showed better results compared to bicycle ergometer training (p<.05). | N/A |
| Cruz et al. (2015)  Case report | Supervised exercise programme (20 m, 2 d/w, 60 min/d) | Aerobic exercise: bicycle ergometer (20 min) (average speed 30 rpm)  Strengthening exercise: stationary resistance equipment - pectoralis major, latissimus dorsi, deltoid, biceps brachii, triceps brachii, abdominal above and below, quadriceps, hamstrings and triceps surae (2 sets/10 reps/45 s rest between sets/10RM with perceived effort of 11 to 13 on the Borg Scale - progression by 20% according to the participant's report) | Improvement in global muscle strength (average overall gain of 121%) and STS (95.74% increase) after the intervention. | One episode |
| Blum (2015)  Systematic review | PubMed, Medline, CINAHL, PEDro and Cochrane (up to 2015): 4 studies (pre-test-post-test) were included. IG: aquatic exercise programmes (4 to 9 w, 2 to 3 d/w). CG: without haemophilia (n=1), unspecified (n=2) | Aquatic exercise: controlled breathing (n=1), stretching (n=2), active movements (n=2), strengthening and Strengthening (n=1), aerobics (n=3) and balance training (n=1) | The findings suggest that aquatic exercise could improve ROM and muscle strength in affected joints, reduce pain and increase aerobic capacity in PwH. However, the results should be considered with caution due to the methodological quality of the included studies. There is a lack of evidence to support aquatic physiotherapy. There is a lack of evidence to support aquatic physiotherapy, therefore studies with larger sample sizes and methodological rigour are required to establish clinical practice guidelines and the ideal dosage for this intervention. | N/A |
| Runkel et al. (2016)  RCT | IG: supervised group exercise programme (36 w, 2 d/w, 90 min/d). CG: usual daily activities | Strengthening exercise + Mobility/ROM exercise + Endurance training (5 to 9 exercises/3 sets/15 reps/10 to 70% rate of perceived effort) | Compared to the CG, IG showed significant improvement in muscle strength (p≤.003), distance covered in 12 minutes (p=.011) and balance on the right side (p=.037) after the intervention. | No increase in bleeding |
| Strike et al. (2016)  Systematic review | Cochrane Cystic Fibrosis and Genetic Disorders Group's Coagulopathies Trials Register (up to December 2016), PubMed, Embase (up to November 2014) and CINAHL (up to April 2014): 8 studies (4 RCTs, 4 quasi-randomised controlled trials) were included. IG: exercise programmes (4 to 12 w, 2 to 5 d/w). CG: no intervention (n=4), intervention with another exercise modality (n=4) | Aquatic exercise (n=1)  Strengthening exercise (n=3)  Static exercise (n=2)  Aerobic exercise (n=3)  Partial weight-bearing exercise (n=1)  Stretching (n=2)  Proprioceptive neuromuscular facilitation (n=1) (n=studies) | Most exercise interventions showed positive effects, with improvements in one or more outcomes, including pain, ROM, strength, and walking tolerance. Aquatic exercises appear to be more effective than land-based exercises in reducing pain in adults, while functional exercises appear to be more effective than static exercises in increasing muscle strength. None of the included studies reported adverse events related to the interventions, including bleeding episodes during exercise. Considering that some of the included studies administered clotting factor prior to the intervention and only included participants with moderate haemophilia, the safety of the exercise modalities evaluated remains uncertain for people with severe haemophilia. There is a lack of confidence in the findings due to the small number of included studies and the inability to pool results due to the heterogeneity of outcomes. | No episodes |
| Schäfer et al. (2016)  Systematic review | PubMed, Web of Science, PEDro, Cochrane, Clinical Trials, SciELO and Lilacs (1980 to May 2014): 9 studies were included. IG: exercise programmes (4 w to 12 m, 1 to 2 d/w). CG: no intervention (n=6), intervention with another exercise programme (n=2), intervention with another physiotherapy modality (n=1) | Strengthening exercise (n=6)  Proprioception exercise (n=2)  Aerobic exercise (n=2)  Aquatic exercise (n=2)  Endurance training (n=2)  Balance training (n=1)  Stretching (n=5) | Overall, the included studies scored low on the JADAD risk of bias scale. The findings suggest that physical exercise and physical therapy techniques can reduce pain perception, as well as improve ROM and muscle strength in PwH. It is recommended that new RCTs be conducted with larger sample sizes and standardisation of intervention and evaluation methods. | N/A |
| Runkel et al. (2017)  RCT | IG: supervised group exercise programme (36 w, 2 d/w, 90 min/d). CG: usual daily activities | Strengthening exercise + Mobility/ROM exercise + Endurance training (5 to 9 exercises/3 sets/15 reps/10 to 70% rate of perceived effort) | Compared to the CG, IG showed significant improvement in the endurance domain of the HEP-Test-Q (p=.000, large effect size), in the general health perception domains (p=.005, large effect size) and mental health ( p=.001, large effect size) of the SF-36, and in the dimensions feeling (p=.049, medium effect size), work (p=.046, medium effect size) and family (p=.04 , large effect size) of Haem-A-QoL after intervention. | N/A |
| Parhampour et al. (2019)  RCT | Supervised exercise programme (6 w, 3 d/w, 40 to 45 min/d). RT: strengthening exercise. AT: aerobic exercise. CT: aerobic + strengthening exercise. CG: no intervention | Strengthening exercise: free weights, stationary resistance equipment - knee extension and flexion, calf raise, leg press, shoulder and chest press, and squats (RT: 10 to 12 reps. CT: 5 to 6 reps/10 s rest between reps/1 to 2 min rest between exercises/65 to 75% 1RM - progression every 2 w)  Aerobic exercise: treadmill and bicycle ergometer - warm-up (AT: 5 min. CT: 3 min), training (AT: 12 min. CT: 6 min) (65 to 75% MHR - progression every 2 w) and cool down (AT: 3 min. CT: 2 min) | Compared to the CG, the RT, AT and CT groups showed a significant reduction (p<.05) in weight, BMI, waist circumference and waist-hip ratio, in addition to an improvement in the total HJHS score (p≤.05). 001) after the intervention. The CT group showed a significant reduction (p≤.02) in high-sensitivity C-reactive protein, interleukin-6 and tumour necrosis factor-α compared to the CG after the intervention. No significant increase in interleukin-10 and adiponectin was observed in the RT, AT and CT groups compared to the CG. | No episodes |
| Neelapala et al. (2019)  Systematic review | PubMed, PEDro, CINAHL and CENTRAL (up to December 2017): 4 studies (2 RCT, 1 pre-test-post-test, 1 cross-sectional) were included. IG: aquatic exercise programmes (3 to 8 w, 3 d/w*). CG: no intervention (n=1) or land exercises (n=1)  *1 study: 1 session of 20 min | Aquatic exercise: stretching (n=1), strengthening and endurance (n=1), active movements (n=2) and running (n=1) (intensity defined in 1 study by MHR) | The methodological quality of the included studies was moderate, with a low level of evidence. Overall, aquatic exercise showed positive effects in PwH, some compared with no intervention and land-based exercise, including improved elbow, knee, and ankle ROM, as well as knee muscle strength. In addition, gains in aerobic capacity and prothrombin time were observed, although with low levels of evidence. Due to limited literature and a lack of studies with robust designs, it was not possible to draw firm conclusions regarding aquatic exercises in PwH. | N/A |
| Deniz & Güzel (2020)  RCT | IG: supervised exercise programme (8 w, 3 d/w) (Phase 1: 0-4 w. Phase 2: 5-8 w) + information on exercise benefits (1 session, 45 min). CG: information on exercise benefits (1 session, 45 min) | Aerobic exercise: treadmill (Phase 1: 10 min at 5 km/h. Phase 2: 15 min at 5 km/h)  Mobility/ROM exercise: shoulder, elbow, hip, knee, ankle and spine (Phase 1 and 2: 10 reps)  Strengthening exercise: elastic bands - deltoid, biceps brachii, triceps brachii, quadriceps, hamstrings, tibialis anterior and triceps surae (Phase 1: 3 sets/10 reps/40% 1RM. Phase 2: 3 sets/15 reps/60% 1RM )  Stretching: posterior shoulder capsule, pectoral, elbow flexors, hamstrings and triceps surae (Phase 1: 3 sets/15 s. Phase 2: 4 sets/20 s)  Balance training: weight-bearing, swinging anteriorposterior-right and left directions (5 reps/15 s duration/ 30 s rest) (Phase 1: floor. Phase 2: BOSU®) | CG had lower levels of kinesiophobia compared to IG before the intervention (p=.033). After the intervention, IG showed a significant decrease in kinesiophobia levels (p=.049) and a significant increase in the SF-36 total score compared to the CG (p=.004). There was no significant increase in the total Hem-A-Qol score of the IG compared to the CG. | No episodes |
| Calatayud et al. (2020)  RCT | IG: supervised group exercise programme (8 w, 2 d/w). CG: usual daily activities | Strengthening exercise: elastic bands - knee (flexion-extension), ankle (plantarflexion-dorsiflexion), elbow (flexion-extension), shoulder (abduction) and hip (abduction) (3 sets/1 repetition below muscle failure, reducing as increasing intensity/1 s concentric contraction/1 s eccentric contraction/1 min rest between sets and exercises/20 to 10RM - progression every 2 w) | Compared to the CG, the IG showed a significant increase in muscle strength in almost all joints (p<.05, medium to large effect size) after the intervention. The IG also presented significantly better scores in TUG (p=.009, medium effect size) and STS (p=.006, medium effect size), greater flexion ROM (ROM) in the knee of the right leg (p =.021, trivial effect size) and better HJHS score in the left knee (p=.027, small effect size). Furthermore, the IG showed a significantly greater reduction in general pain status (p=.037), better self-assessment of general status (p<.001) and greater desire to exercise (p<.001) compared to the CG. | No episodes |
| Wagner et al. (2020)  Systematic review | PubMed, Scopus, Medline, CINAHL, Cochrane and PEDro (1960 to November 2019): 14 studies (9 RCTs, 1 controlled trial, 4 single-arm prospective studies) were included. IG: exercise programmes (6 w to 2 y, 2 to 7 d/w). CG: no intervention (n=7), intervention with another modality of exercise/physiotherapy (n=3), unspecified (n=4) | Strengthening exercise: dynamic (n=6), isometric (n=1), isokinetic (n=1), isometric + dynamic (n=5), isometric + dynamic + isokinetic (n=1)  Only 3 studies applied exercise programmes exclusively with strengthening exercise. The remaining studies applied multimodal exercise programmes, including proprioception/coordination, endurance, flexibility and relaxation. | The findings suggest that training intensities lower than those recommended for increasing strength in healthy people are effective for increasing strength in PwH. None of the included studies reported bleeding episodes related to the interventions. Resistance exercise appears to be a safe intervention when properly supervised, individually adapted and employed in association with factor therapy, although the optimal dosage (frequency, intensity, and duration) has not yet been determined. The findings should be considered with caution due to the methodological quality of the included studies and the inability to group the results based on the heterogeneity of the designs, interventions and outcomes. | No episodes |
| García-Dasí et al. (2021)  Two-arm prospective | IG: home-based exercise programme (16 w, 3 d/w, 60 min/d) + CBT intervention (4 monthly individual sessions, 120 min). CG: usual daily activities | Mobility/ROM exercise: ankles, knees, shoulders, elbows, wrists and hips (1 set/10 reps)  Aerobic exercise: walking (initial intensity 3-4 on OMNI-GSE)  Strengthening exercise: elastic bands - knees (flexion and extension), ankles (plantar flexion), hips (abduction), elbows (flexion and extension) and shoulders (abduction) (3 sets/10 reps/1 min rest/initial intensity 3- 4 on OMNI-RES)  Stretching: pectoralis major, latissimus dorsi, trapezius, quadriceps and hamstrings (30 s) | IG showed significant improvement in the symptom control (p=.038) and pain management (p=.005) dimensions of the Chronic Pain Self-Efficacy Scale, in quality of life (p<.001), in self-esteem (p=.001). 033), pain (p<.001) and kinesiophobia (p<.001) compared to the CG after the intervention. The effects of the intervention remained significant over time in the IG for pain management (p<.001), quality of life (p<.001), pain (p<.001) and kinesiophobia (p<.001). | N/A |
| Parhampour et al. (2021)  RCT | Supervised exercise programme (6 w, 3 d/w, 45 min/d). RT: strengthening exercise. AT: aerobic exercise. CT: aerobic + strengthening exercise. CG: no intervention | Stretching: pre-exercise warm-up (10 min)  Strengthening exercise: free weights, stationary resistance equipment - squats, leg press, chest and shoulder press, knee flexion and extension, and calf raise (RT: 10 to 12 reps. CT: 5 to 6 reps/10 s rest between reps /1 to 2 min rest between exercises/65 to 75% 1RM - progression every 2 w)  Aerobic exercise: treadmill, bicycle ergometer - warm-up (AT: 5 min. CT: 3 min), training (AT: 12 min. CT: 6 min) (65 to 75% MHR - progression every 2 w) and cool down (AT: 3 min. CT: 2 min) | Compared to the CG, the RT, AT and CT groups showed a significant reduction in weight, BMI, waist circumference, waist-hip ratio, LDL-C and total cholesterol (p<.05), in addition to an increase significant in HDL-C and quality of life (p<.001) after the intervention. No significant differences were observed between the RT, AT and CT groups after the intervention. However, compared to control, a more significant improvement in lipid profile and quality of life was observed with combined training compared to resistance training and aerobic training. | No episodes |
| Pillard et al. (2021)  Exploratory | Home-based endurance training programme (6 w, 3 d/w, 45 min/d) | Endurance training: bicycle ergometer - warm-up (5 min), interval training (1 min at high intensity/5 min recovery period/4 min at moderate intensity) (personalised intensity, based on the power and HR values ​​achieved in the 1st and 2nd ventilatory threshold, respectively, in an exercise test) | Significant improvement in endurance power [maximum aerobic power (p=.003); power at the first ventilatory threshold (p=.0006); power at the second ventilatory threshold (p=.0004)] after the intervention. In participants with mild haemophilia, endurance training slightly increased FVIII levels compared to rest (p<.05). | No episodes |
| Parhampour et al. (2022)  RCT | Supervised exercise programme (6 w, 3 d/w, 40 to 45 min/d). RT: strengthening exercise. CT: aerobic + strengthening exercise. CG: no intervention | Stretching: pre-exercise warm-up (10 min)  Strengthening exercise: free weights, stationary resistance equipment - squats, leg press, chest and shoulder press, knee flexion and extension, and calf raise (RT: 10 to 12 reps. CT: 5 to 6 reps/10 s rest between reps /1 to 2 min rest between exercises/65 to 75% 1RM - progression every 2 w)  Aerobic exercise: treadmill, bicycle ergometer - warm-up (3 min), training (6 min) (65 to 75% MHR - progression every 2 w) and cool-down (2 min) | Compared to CG, the RT and CT groups showed significant improvement (p<.001) in the muscle thickness of the vastus medialis, vastus lateralis, gastrocnemius medialis, biceps brachii and triceps brachii, and in the pennation angle of the vastus medialis, vastus lateralis and gastrocnemius medial after the intervention. No significant differences were observed between the RT and CT groups after the intervention. | No episodes |
| Gönen et al. (2022)  RCT | Supervised exercise programme (12 w, 2 d/w) IG: closed kinetic chain. CTG: as described in the literature. CG: no intervention | IG: Strengthening exercises (body weight, elastic bands) and balance (↓ BoS, balance board) in a closed kinetic chain for the lower limbs (10 reps/5 s contraction/5 s rest between reps)  CTG: Mobility/ROM exercises (hip, knee and ankle) (2 sets/10 reps/5 s rest between reps), isometric and isotonic strengthening (body weight, free weights, elastic bands - hip, ankle and knee) (10 reps/ 5 s contraction/5 s rest between reps/40 to 60% MVIC), balance and proprioceptive | A significant difference was observed between the groups in proprioception (p=.001) and physical activity level [TUG (p=.008), STS (p=.001), FISH (p=.006)] after the intervention. The IG showed a significantly greater improvement (p<.05) in the evaluated outcomes compared to the other two groups. | N/A |
| Fares et al. (2022)  RCT | Both: supervised exercise programme (8 w, 3 d/w). IG: aerobic training (30 min/d) | Mobilisation: spine, hip, knee, ankle, shoulder, elbow and/or wrist (depending on the patient's condition) (10 min)  Balance training: bipedal support with reduced base, tandem, single-leg support and single-leg support associated with movement of the contra-lateral limb  Strengthening exercise: unspecified  Flexibility exercise: not specified  Aerobic exercise: bicycle ergometer - warm-up (5 min), pedalling with gradual increase in load at moderate intensity (50 to 60% MHR) and cool-down (5 min) | Significant improvement (p<.05) in IG lung function in all variables evaluated after the intervention. Compared to the CG, IG showed significant improvement in all variables measured after the intervention (p<.05). | N/A |
| Tomschi et al. (2022)  Systematic review | PubMed and Web of Science (January 2000 to May 2021): 7 studies (3 RCTs, 2 controlled trials, 2 prospective single-arm studies) were included. IG: aerobic exercise programmes (6 to 12 w, 1 to 3 d/w). CG: no intervention (n=27) or intervention with another exercise modality (n=75) | Aerobic exercise: Nordic walking (n=1), treadmill (n=2), bicycle ergometer associated with treadmill (n=1) and aquatic exercise (n=3) (intensity defined by HR or Borg***) ***2 studies: N/A | High heterogeneity was observed in the methodological quality of the included studies. Six of the seven studies did not report any adverse events related to the aerobic exercise intervention, such as bleeding episodes during exercise, increased bleeding frequency, or increased factor consumption. Aerobic exercise has shown positive effects in PwH, including improvements in cardiovascular performance, joint health, muscle strength, anthropometric and inflammatory parameters, and bone mineral density. Therefore, aerobic exercise can be considered a viable and safe option for PwH when supervised by experts. | No episodes or increased bleeding |
| Chimeno-Hernández et al. (2022)  Systematic review | PubMed, Web of Science, Embase, Wiley Online Library and CENTRAL (up to November 2021): 10 studies (4 RCT, 3 pre-test-post-tests with 2 CG, 3 pre-test-post-tests without CG) were included. IG: exercise programmes (1 to 13 m, 2 to 7 d/w). CG: no intervention (n=3), intervention with another exercise modality (n=1), intervention with another physiotherapy modality (n=2) | Strengthening and Balance training (n=10)  Flexibility exercise (n=5)  Mobility/ROM exercise (n=2)  Flexibility and mobility/ROM exercise (n=1)  Aerobic exercise (n=1)  Gait training (n=3) | In general, the included studies presented a reasonable risk of bias, with a very low certainty of evidence. Five out of the ten included studies, which incorporated strengthening, balance, flexibility, and aerobic exercises, showed significant improvements in balance after the interventions. The findings indicate that the evidence on the use of exercise to improve balance PwH IS unclear. | N/A |
| Cruz-Montecinos et al. (2023)  RCT | IG: supervised exercise programme (8 w, 2 d/w). CG: usual daily activities | Strengthening exercise: elastic bands - knees, elbows and ankles (3 sets/1 s concentric contraction/1 s eccentric contraction/1 min rest/20 to 10RM - increasing intensity every 2 w) | IG showed significant improvement in the joint damage dimension of the A36 haemophilia-QoL (p=.015, large effect size), and in the lying, sitting, kneeling and standing dimensions (p=.006, small effect size) and complex lower limb activities in HAL (p=.006, small effect size) compared to the CG. No other significant differences were observed. | No episodes |
| Moreno-Segura et al. (2023)  RCT | IG: home-based exercise programme (16 w, 3 d/w) + CBT intervention (4 monthly individual sessions). CG: usual daily activities | Mobility/ROM exercise: ankles (flexion-extension), knees (flexion-extension), hips (circumduction), shoulders (circumduction), elbows (flexion-extension) and wrists (circumduction) (1 set/10 reps)  Aerobic exercise: walking (20 to 30 min) (intensity 3-4 to 6-7 on the OMNI-GSE)  Strengthening exercise: elastic bands - knees (flexion and extension), ankles (plantar flexion), hips (abduction), elbows (flexion and extension) and shoulders (abduction) (3 sets/10 reps/1 min rest/intensity 3-4 to 6-7 in OMNI-RES)  Stretching: pectoralis major, latissimus dorsi, trapezius, quadriceps and hamstrings (1 set/2 reps/30 s) | IG showed significant improvement in the dimensions of function (p<.001), pain (p<.001), joint damage (p=.006) and satisfaction with treatment (p=.006) of the A36 haemophilia-QoL, as well as in pain measured with VAS (p=.008) and PainDETECT (p=.035) after the intervention. No other significant intragroup differences were observed. | N/A |
| Wilczyński et al. (2023)  Case report | Supervised exercise programme (6 w, 3 d/w, 60 min/d) | Strengthening exercise: trunk, lower limbs and upper limbs (7 exercises) (4 sets/8 to 20 reps/90 s and 60 s rest between exercises and sets, respectively/80% 20 RM) | Improvements in quality of life, specifically in the health and function domain of the QLI - Generic III Version, and in the power of the lower limbs (MDC 3.9) after the intervention. No musculoskeletal injuries or bleeding episodes were observed during the intervention and after 3 months of follow-up. | No episodes |
| Elnaggar et al. (2024)  RCT | IG: supervised aquatic plyometric exercise programme (12 w, 2 d/w, 45 min/d) (W1&4: 1-4 w. W5&8: 5-8 w. W9&12: 9-12 w). CG: supervised exercise programme (12 w, 45 min/d) | IG: Aerobic exercise (bicycle ergometer or treadmill (50% FCM), active upper limb movements associated with breathing and free running in the water) (10 minutes) + Aquatic plyometric exercise (10 exercises for lower limbs - intensity determined through a pilot test, maximum possible effort encouraged) (25 to 30 min) (W1&4: 1 to 3 sets/3 to 10 reps/1 to 2 min rest. W5&8: 1 to 3 sets/3 to 15 reps/1 to 2 min rest. W9&12: 2 to 5 sets/5 to 10 reps/1 to 2 min rest) + Stretching (5 min)  GC: Stretching + Mobility/ROM exercise + Strengthening exercise (isometric with progression to low-intensity dynamic with elastic bands) + Balance training (static and dynamic, unspecified) + Proprioceptive exercise (leg-press or multidirectional steps) + Aerobic exercise (bicycle ergometer or treadmill) (according to tolerance) | Compared to the CG, IG showed a significant improvement in pain (p=.028), in quadriceps muscle strength [120°/s (p=.007); 180°/s (p=.011)] and hamstrings [120°/s (p=.024); 180°/s (p=.036)], in directional postural stability [forward (p=.007); backwards (p=.013); affected side (p=.008); unaffected side (p=.002)] and global (p<.001), and functional capacity [FISH (p<.001); 6MWT (p=.002)] after intervention. | N/A |
| Srichumpuang et al. (2024)  Non-RCT | Individualised exercise programme (24 w, 7 d/w). 1: young children with 0 to 1 target joint. 2: adolescents with 2 to 3 target joints. 3: young adults with >3 target joints | 1: Stretching + Strengthening exercise (body weight - squats, bridge, calf raises and plank) (1 d/week, 30 min/d) + Mobility/ROM exercise (1 d/week, 30 min/d) + Balance training (1 d/week, 30 min/d) + Aerobic exercise (free activity) (3 d/week) - intensity aiming for a 65% increase in MHR  2 and 3: Stretching + Strengthening exercise (body weight - squats, bridges, calf raises, planks, wall push-ups and hamstring curls) (6 sets/15 s) (30 to 45 min/d) + Endurance training (treadmill, body weight - 3 sets/10 min) (1 d/week, 30 to 45 min/d) + Balance training (3 sets/1 min) (1 d/week, 30 to 45 min /d) + Aerobic exercise (free activity) (3 d/week) - intensity aiming for a 65% increase in MHR | Significant improvement in annual bleeding rate (p<.05), annual joint bleeding rate (p<.05), HJHS score (p<.05), muscle mass (p=.001), quality of life (p=.001) and annual factor consumption (p=.001) after the intervention. | No joint bleeds in 46.2% of the patients |

Abbreviations: 6MWT, Six Minute Walk Test; A36 Hemophilia-QoL, Hemophilia-specific health-related quality of life questionnaire; BMI, body mass index; BoS, base of support; CTG, conventional treatment group; FISH, Functional Independence Score in Hemophilia; GRADE, Grading of Recommendations Assessment, Development, and Evaluation; HAL, Hemophilia Activities List; Haemo-QoL, Quality of life assessment instrument for children and adolescents with haemophilia; Hem-A-QoL, Haemophilia Quality of Life Questionnaire for Adults; HDL-C, high-density lipoprotein cholesterol; HEP-Test-Q, Questionnaire for the assessment of subjective physical functioning; HJHS, Hemophilia Joint Health Score; LDL-C, low-density lipoprotein cholesterol; MDC, Minimal Detectable Change; MHR, maximum heart rate; min, minutes; MVIC, maximum voluntary isometric contraction; QLI, Quality of Life Index; RCTs, randomised clinical trials; RM, repetition maximum; ROM, range of motion; s, seconds; SF-36, Short Form Health Survey; STS, Sit To Stand Test; TUG, Timed Up and Go.
